# Inhibition of dihydrotestosterone synthesis in prostate cancer by combined frontdoor and backdoor pathway blockade

**DOI:** 10.18632/oncotarget.24107

**Published:** 2018-01-10

**Authors:** Michael V. Fiandalo, John J. Stocking, Elena A. Pop, John H. Wilton, Krystin M. Mantione, Yun Li, Kristopher M. Attwood, Gissou Azabdaftari, Yue Wu, David S. Watt, Elizabeth M. Wilson, James L. Mohler

**Affiliations:** ^1^ Department of Urology, Roswell Park Cancer Institute, Buffalo, NY 14263, USA; ^2^ Department of Pharmacology and Therapeutics, Roswell Park Cancer Institute, Buffalo, NY 14263, USA; ^3^ Department of Biostatistics and Bioinformatics Roswell Park Cancer Institute, Buffalo, NY 14263, USA; ^4^ Department of Pathology, Roswell Park Cancer Institute, Buffalo, NY 14263, USA; ^5^ Center for Pharmaceutical Research and Innovation and Department of Molecular and Cellular Biochemistry, University of Kentucky, Lexington, KY 40536, USA; ^6^ Department of Biochemistry and Biophysics, University of North Carolina, Chapel Hill, NC 27599, USA

**Keywords:** androstanediol, dihydrotestosterone, dutasteride, 3α-oxidoreductases, androgen deprivation therapy

## Abstract

Androgen deprivation therapy (ADT) is palliative and prostate cancer (CaP) recurs as lethal castration-recurrent/resistant CaP (CRPC). One mechanism that provides CaP resistance to ADT is primary backdoor androgen metabolism, which uses up to four 3α-oxidoreductases to convert 5α-androstane-3α,17β-diol (DIOL) to dihydrotestosterone (DHT). The goal was to determine whether inhibition of 3α-oxidoreductase activity decreased conversion of DIOL to DHT. Protein sequence analysis showed that the four 3α-oxidoreductases have identical catalytic amino acid residues. Mass spectrometry data showed combined treatment using catalytically inactive 3α-oxidoreductase mutants and the 5α-reductase inhibitor, dutasteride, decreased DHT levels in CaP cells better than dutasteride alone. Combined blockade of frontdoor and backdoor pathways of DHT synthesis provides a therapeutic strategy to inhibit CRPC development and growth.

## INTRODUCTION

Prostate cancer (CaP) growth and progression rely on the activation of the androgen receptor (AR) by the testicular androgen, testosterone (T), or its more potent metabolite, dihydrotestosterone (DHT) [1]. Almost all men who present with advanced CaP and some men who fail potentially curative therapy are treated with androgen deprivation therapy (ADT). ADT lowers circulating T levels, deprives AR of ligand and induces CaP regression [2, 3]; however, ADT is only a temporary palliative measure. After ADT, intratumoral androgen levels remain sufficient to activate AR [1, 4] and CaP recurs as lethal, castration-recurrent/resistant CaP (CRPC).

**Figure 1:**
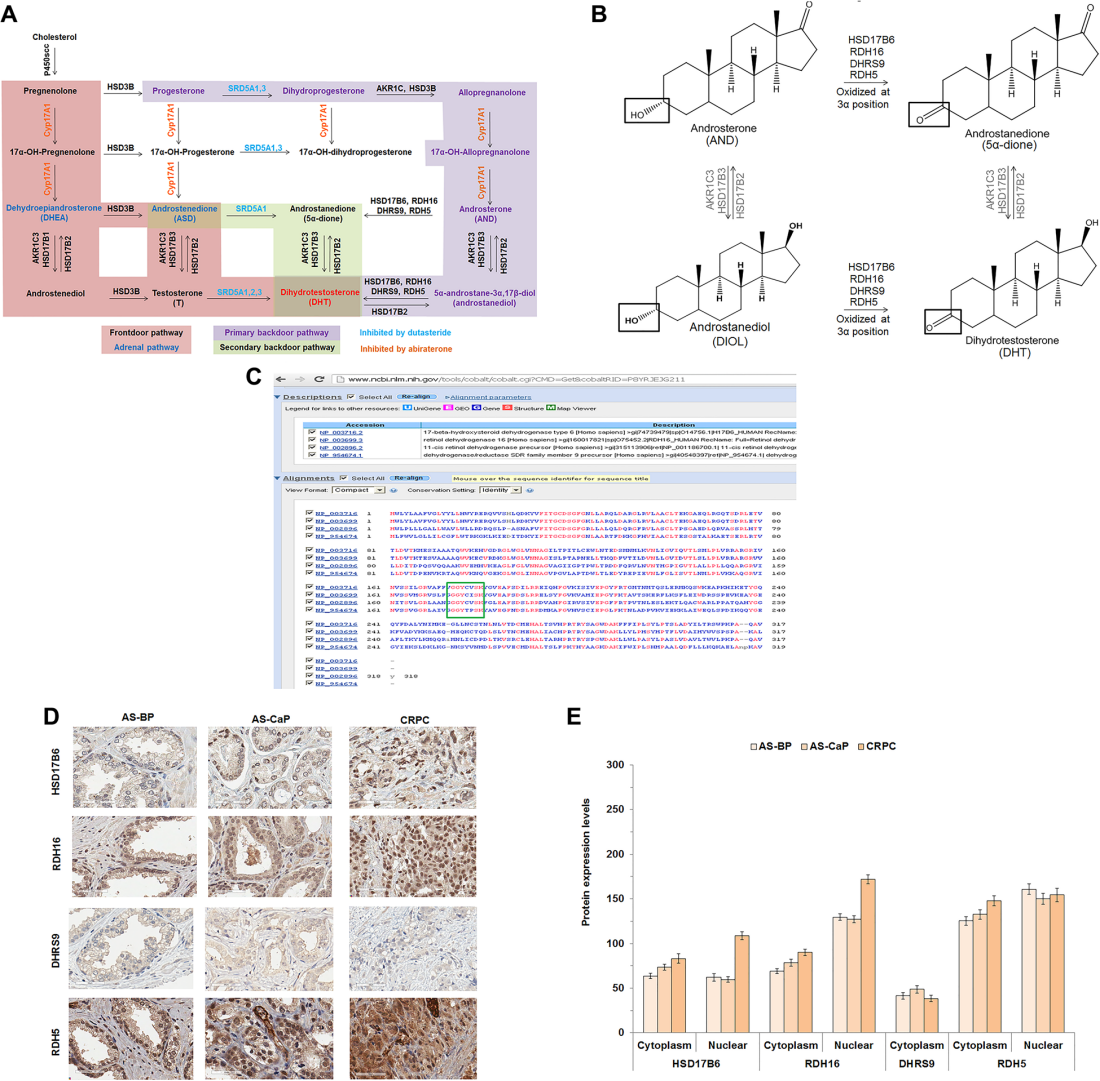
HSD17B6, RDH16, DHRS9 and RDH5 expression in AS-BP, AS-CaP and CRPC Androgen metabolism pathways for DHT synthesis (**A**). Diagram of 3α-oxidoreductase activity (**B**). COBALT 3α-oxidoreductase protein sequence alignment with the catalytic site highlighted (green box) (**C**). IHC of four endogenous 3α-oxidoreductases in AS-BP, AS-CaP and CRPC (**D**). Positive and negative control images were shown in Supplementary Figure 1. Scores showed that HSD17B6 and RDH16 were expressed at higher levels in CRPC than ASBP or AS-CaP tissue (**E**). Data were presented as the mean +/- SEM. Protein expression was modeled as a function of tissue type (AS-BP, AS-CaP or CRCP), compartment (cytosol or nuclear), their interaction, and a random rater effect using a linear mixed model (LMM). Mean expression was compared among tissue types and between compartments using Tukey-Kramer adjusted tests about the least square means. *P-*values were listed in Supplementary Table 4.

One mechanism that may contribute to CaP resistance to ADT is intratumoral DHT synthesis from adrenal androgens or T [5]. Three androgen pathways produce DHT (Figure 1A, modified from [6]) from three different, penultimate precursors: reduction of the ∆^4^-double bond in T by 5α-reductase (SRD5A) 1, 2 or 3; reduction of the 17-keto group in 5α-androstane-3,17-dione (5α-dione) by ARK1C3 or HSD17B3; or oxidation of the 3α-hydroxyl group in 5α-androstane-3α,17β-diol (DIOL) by 3α-oxidoreductases. The frontdoor pathway uses the adrenal androgens, dehydroepiandrosterone (DHEA) or 4-androstene-3,17-dione (ASD), as precursors to generate T that undergoes reduction to DHT by SRD5A 1, 2 or 3 [7]. Two backdoor pathways generate DHT without using T as an intermediate. The primary and secondary backdoor pathways convert DIOL or 5α-dione, respectively, to DHT (Figure 1A).

Previous work from our laboratory and others demonstrated that CaP cells use both primary and secondary backdoor pathways to synthesize DHT [5, 8–10]. The terminal step in the primary backdoor pathway is the conversion of DIOL to DHT [5, 10, 11] by any of the four 3α-oxidoreductases: [1] 17β-hydroxysteroid dehydrogenase (HSD) 6 (HSD17B6); [2] retinol dehydrogenase (RDH) 16 (RDH16); [3] dehydrogenase/reductase family member 9 (DHRS9, formerly RDH15); and [4] dehydrogenase/reductase family member 5 (RDH5) [5, 11–13]. The secondary backdoor pathway involves the conversion of DHEA to ASD by HSD3B1 or HSD3B2. ASD is converted subsequently to 5α-dione by SRD5A1 and 5α-dione is converted to DHT by AKR1C3 or HSD17B3 [9, 14–16].

Androgen metabolism inhibitors, such as the SRD5A inhibitor, dutasteride, or the CYP17A1 inhibitor, abiraterone [7, 17], have been disappointing clinically. Dutasteride inhibited SRD5A activity [18], depressed T uptake and lowered DHT levels *in vitro* [19], but dutasteride proved ineffective against CRPC in a Phase II clinical trial [20]. CYP17A1 metabolized steroids, such as pregnenolone or progesterone to adrenal androgens, such as DHEA, that provided intermediates for the androgen pathways that generated T and DHT [21]. Abiraterone decreased intratumoral DHT levels [22], but abiraterone extended survival by only approximately 4 months [23]. CaP resistance to abiraterone presumably resulted from enzyme redundancy, progesterone accumulation that led to increased CYP17A1 expression and/or the generation of AR splice variants [24–27]. The need to produce an androgen metabolism inhibitor that performs better than abiraterone has become more important since abiraterone will become used earlier in the disease as a result of the demonstration of improved survival when used with standard ADT for newly diagnosed metastatic CaP [28, 29].

No inhibitors are available clinically to block the conversion of DIOL to DHT by 3α-oxidoreductases. In this report, we show that the catalytic activity of the 3α-oxidoreductases is critical for metabolism of 5α-androstan-3α-ol-17-one (androsterone; AND) to 5α-dione and DIOL to DHT. Inhibition of the terminal steps of the frontdoor pathway using dutasteride and the primary backdoor pathway using 3α-oxidoreductase catalytic mutants lowered DHT more effectively than either alone.

## RESULTS

### 3α-oxidoreductase enzymes shared a conserved catalytic site

The primary backdoor pathway uses one or more of four 3α-oxidoreductases to convert DIOL to DHT or AND to 5α-dione (Figure 1B). DHT synthesis from adrenal androgens was suggested to contribute to the development and growth of CRPC [5, 8, 10, 30]. Inhibition of a single 3α-oxidoreductase enzyme could fail due to enzyme redundancy and/or expression of more than one enzyme. Therefore, an optimal therapeutic approach is to inhibit all four 3α-oxidoreductases. Constraint-based Multiple Protein Alignment Tool (COBALT) protein sequence analysis showed that the four 3α-oxidoreductases shared a common catalytic site (Figure 1C).

### HSD17B6, RDH16, DHRS9 and RDH5 were expressed in clinical CaP

Analysis of immunohistochemistry (IHC) performed on Tissue Micro Array sections from a total of 72 patients showed that HSD17B6, RDH16, DHRS9 and RDH5 were expressed in androgen-stimulated (AS) benign prostate (BP), AS-CaP and CRPC (Figure 1D). DHRS9 was expressed only in the cytoplasm. Nuclear expression levels of HSD17B6 and RDH16, but not RDH5, were higher in CRPC tissues than in AS-BP or AS-CaP tissues (Figure 1E; Supplementary Table 4). RDH16 levels were higher in the nucleus than the cytoplasm (Figure 1E; Supplementary Table 4). Peri-nuclear enhancement was observed for each 3α-oxidoreductase except DHRS9, in AS-BP, AS-CaP and CRPC tissues.

### 3α-oxidoreductase gene expression varied among CaP cell lines

**Figure 2:**
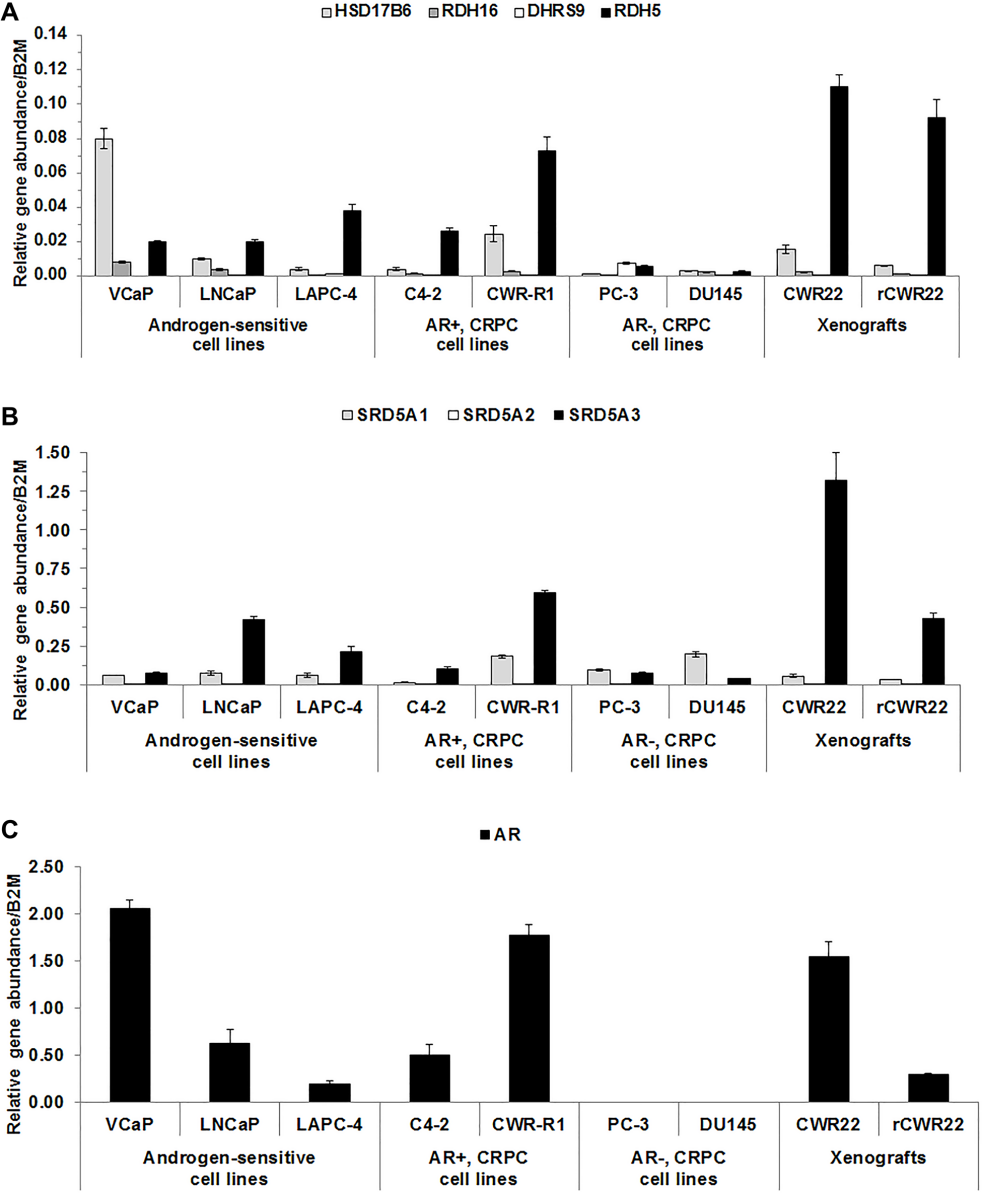
3α-oxidoreductases were expressed in CaP cell lines and xenografts qRT-PCR results were shown for 3α-oxidoreductase (**A**), SRD5A (**B**), and AR (**C**) mRNA levels for CaP cell lines and CWR22 and rCWR22 xenografts. Data were presented as mean +/- SEM. Gene expression was modeled as a function of cell line and a random replicate effect using a LMM. Mean expression was compared among cell lines using Tukey-Kramer adjusted tests about the least square means.

Although 3α-oxidoreductases were detected in clinical samples using IHC, they were not detectable in CaP cell lines using western blot analysis. Therefore, quantitative real-time polymerase chain reaction (qRT-PCR) was performed to determine 3α-oxidoreductase, SRD5A and AR gene expression profiles in CaP cell lines and the androgen-dependent human CWR22 and castration-recurrent CWR22 (rCWR22) CaP xenografts. RDH5 mRNA levels were higher than the expression of the other three 3α-oxidoreductases in all cell lines, except VCaP, PC-3 and DU145 (Figure 2A). SRD5A3 mRNA levels were higher than SRD5A1 in all cell lines, except PC-3 and DU145 (Figure 2B). SRD5A2 mRNA was not measurable, which is consistent with reports of low SRD5A2 gene expression in CaP cell lines [19] and clinical specimens [31]. AR mRNA was expressed in all CaP cell lines, except PC-3 and DU145, and in both xenografts (Figure 2C) [32, 33]. The data suggested that analysis of 3α-oxidoreductase activity in human CaP cell lines required transient expression.

### DHT levels increased when RDH16, DHRS9 or RDH5 were expressed in LAPC-4 cells

The effect of 3α-oxidoreductase expression on DHT levels was determined in the androgen-sensitive LAPC-4 cell line that expressed wild-type AR and RDH5 (Figure 2A) and exhibited 5α-reductase activity [19]. VCaP and LNCaP-RPCI (LNCaP) cells [19] were not used initially because conversion of T to DHT was not detected. Wild-type 3α-oxidoreductases were expressed transiently in LAPC-4 cells. Results were compared to LAPC-4 cells transfected with empty plasmid, which were expected to have low endogenous 3α-oxidoreductase activity based on endogenous RDH5 in LAPC-4 cells (Figure 2A). LAPC-4 cells were treated with DIOL, AND or T. DIOL and AND were used for treatment because 3α-oxidoreductases convert DIOL to DHT or AND to 5α-dione (Figure 1B) [5, 12, 13]. T treatment was used as a control condition because 3α-oxidoreductases do not convert T to DHT [12, 13].

**Figure 3:**
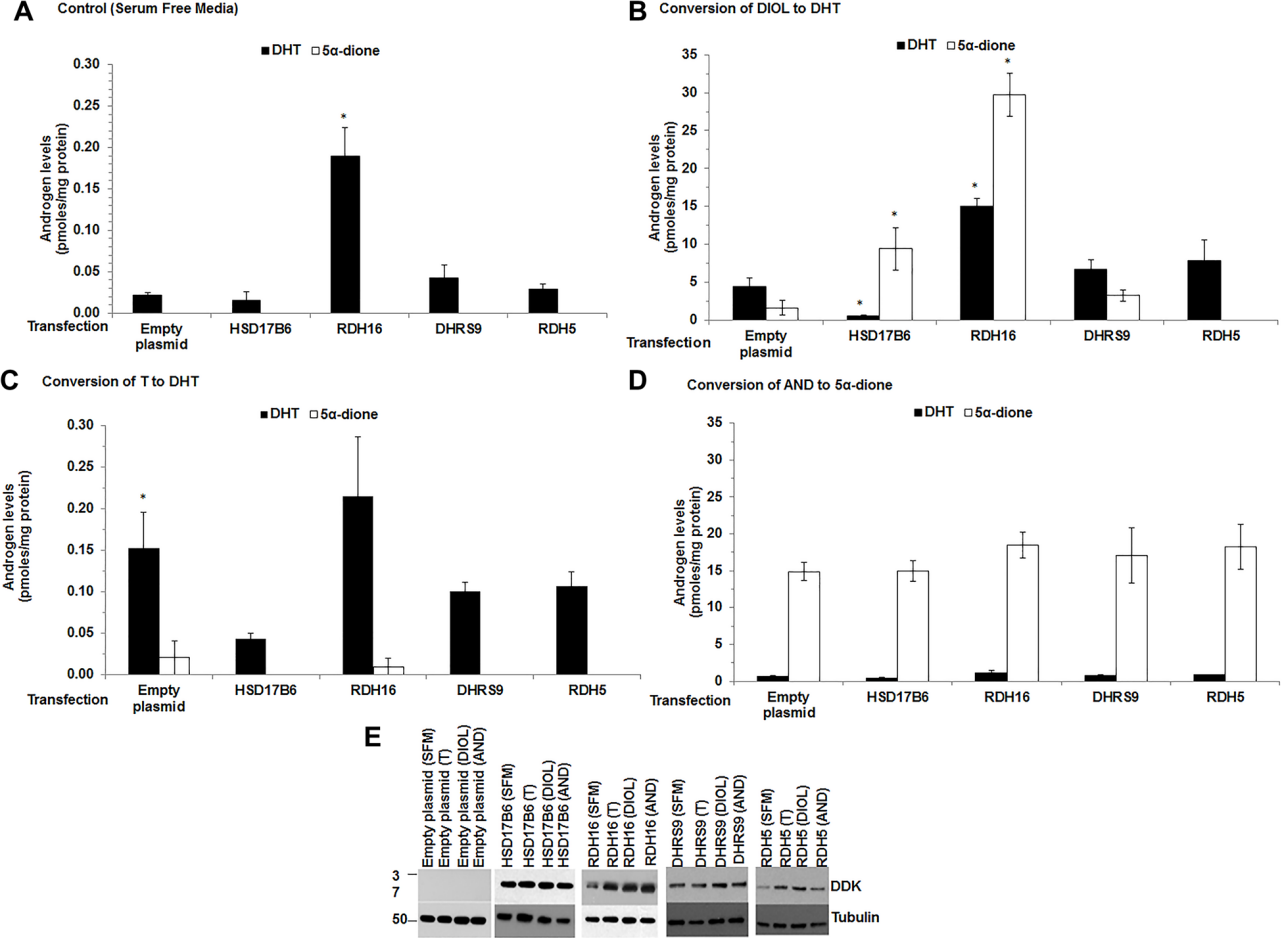
DHT levels increased when RDH16 was expressed in LAPC-4 cells Androgen levels were measured using LC-MS/MS from media and cell pellets of LAPC-4 cells transfected with empty plasmid or expression plasmids encoded with HSD17B6, RDH16, DHRS9 or RDH5. Media and cell pellet androgen levels were combined. Cells were treated for 12 h in SFM alone (**A**) or SFM with 20 nM DIOL (**B**) or 1 nM T (**C**) or 20 nM AND (**D**). Western blot analysis using DDK antibody was used to confirm enzyme expression (E). Western blot images were cropped to conserve presentation space. Data were presented as mean +/- SEM. Androgen levels were modeled as a function of treatment (SFM, DIOL, T or AND), enzyme (HSD17B6, RDH16, DHRS9 or RDH5), and their interaction using a general linear model (GLM). Mean androgen levels were compared among DIOL, T or AND treated LAPC-4 cells against SFM treated LAPC-4 cells for each analyte. Comparisons were made among LAPC-4 cells that expressed each 3α-oxidoreductase and LAPC-4 cells with empty plasmid for each treatment using Dunnett adjusted post-hoc tests. *P-*values were reported in Supplementary Table 6. ^*^p < 0.05.

Liquid chromatography-tandem mass spectrometry (LC-MS/MS) analysis revealed that LAPC-4 cells transfected with empty plasmid in Serum Free Complete Media (SFM; medium without exogenous androgen) produced low levels of DHT (0.0211 pmoles/mg protein) (Figure 3A empty plasmid). This finding was consistent with qRT-PCR data (Figure 2A) and previous reports [5] that LAPC-4 cells expressed endogenous RDH5 that metabolized DIOL to DHT. DHT levels were higher (*p* = 0.008) in SFM treated LAPC-4 cells that expressed RDH16 compared to SFM treated LAPC-4 cells with empty plasmid (Figure 3A [RDH16 vs. empty plasmid] and Supplementary Table 6), which suggested that RDH16 enhanced DHT synthesis in LAPC-4 cells.

LAPC-4 cells with empty plasmid treated with DIOL produced higher levels of DHT compared to SFM treated LAPC-4 cells with empty plasmid (Figure 3B [note the change in Y axis between panels A and B]; Supplementary Table 6). DIOL treated LAPC-4 cells that expressed RDH16 produced higher (*p* = 0.022) levels of DHT than SFM treated LAPC-4 cells with empty plasmid (Figure 3A and Supplementary Table 6). DIOL treated LAPC-4 cells that expressed HSD17B6 produced lower DHT levels (*p* < 0.001) compared to LAPC-4 cells with empty plasmid, which suggested that LAPC-4 cells may possess inhibitory mechanisms that impaired HSD17B6 activity.

DHT levels were higher (*p* = 0.024) when LAPC-4 cells with empty plasmid were treated with T (Figure 3C and Supplementary Table 6) compared to SFM treated LAPC-4 cells with empty plasmid, which is consistent with T to DHT conversion by endogenous SRD5A [19]. DHT levels were not significantly different among T treated LAPC-4 cells that expressed 3α-oxidoreductases or LAPC-4 cells with empty plasmid (Supplementary Table 6).

5α-dione was produced when LAPC-4 cells with empty plasmid were treated with DIOL or AND (Figure 3B). The data were consistent with the levels of endogenous RDH5 mRNA found in LAPC-4 cells (Figure 2A). DIOL treated LAPC-4 cells that transiently expressed HSD17B6, RDH16 or DHRS9 produced 5α-dione, but only LAPC-4 cells that expressed HSD17B6 (*p* = 0.022) or RDH16 (*p* = 0.003) produced higher 5α-dione levels compared to LAPC-4 cells with empty plasmid (Figure 3B; Supplementary Table 6). The data suggested LAPC-4 cells endogenously converted DIOL to DHT and HSD17B6 or RDH16 converted AND to 5α-dione. AND treated LAPC-4 cells that transiently expressed 3α-oxidoreductases produced similar 5α-dione levels as AND treated LAPC-4 cells with empty plasmid (Figure 3D). The data suggested that LAPC-4 cells may not be an appropriate CaP cell model to study the ability of 3α-oxdioredcutases to convert AND to 5α-dione.

Taken together, these data were consistent with reports that 3α-oxidoreductases metabolize DIOL to DHT or AND to 5α-dione [5, 10, 12, 13].

### Catalytic amino acid substitution or deletion impaired 3α-oxidoreductase activity

Previous reports showed that 3α-oxidoreductases contained a catalytic tyrosine (Y) and lysine (K) that lowered the binding energy requirements and promoted the metabolism of androgen precursors [12, 13, 34]. Site-directed mutagenesis of the 3α-oxidoreductase catalytic residues confirmed Y176/175 and K179/180 were essential for 3α-oxidoreductase activity for all four enzymes. The mutants included ∆cat (deletion of the catalytic residues) or double mutants, Y→F, K→R (Y176F for HSD17B6, RDH16, DHRS9 or Y175F for RDH5 and K180R for HSD17B6, RDH16 and DHRS9 or K179R for RDH5). Rationale for the Y→f mutation rested on their similarity in size but difference in an essential hydroxyl group. This mutation was not expected to alter protein folding but would affect enzyme activity. The K→R mutation was chosen because R would maintain a positive charge but was not expected to alter protein folding [34, 35].

**Figure 4:**
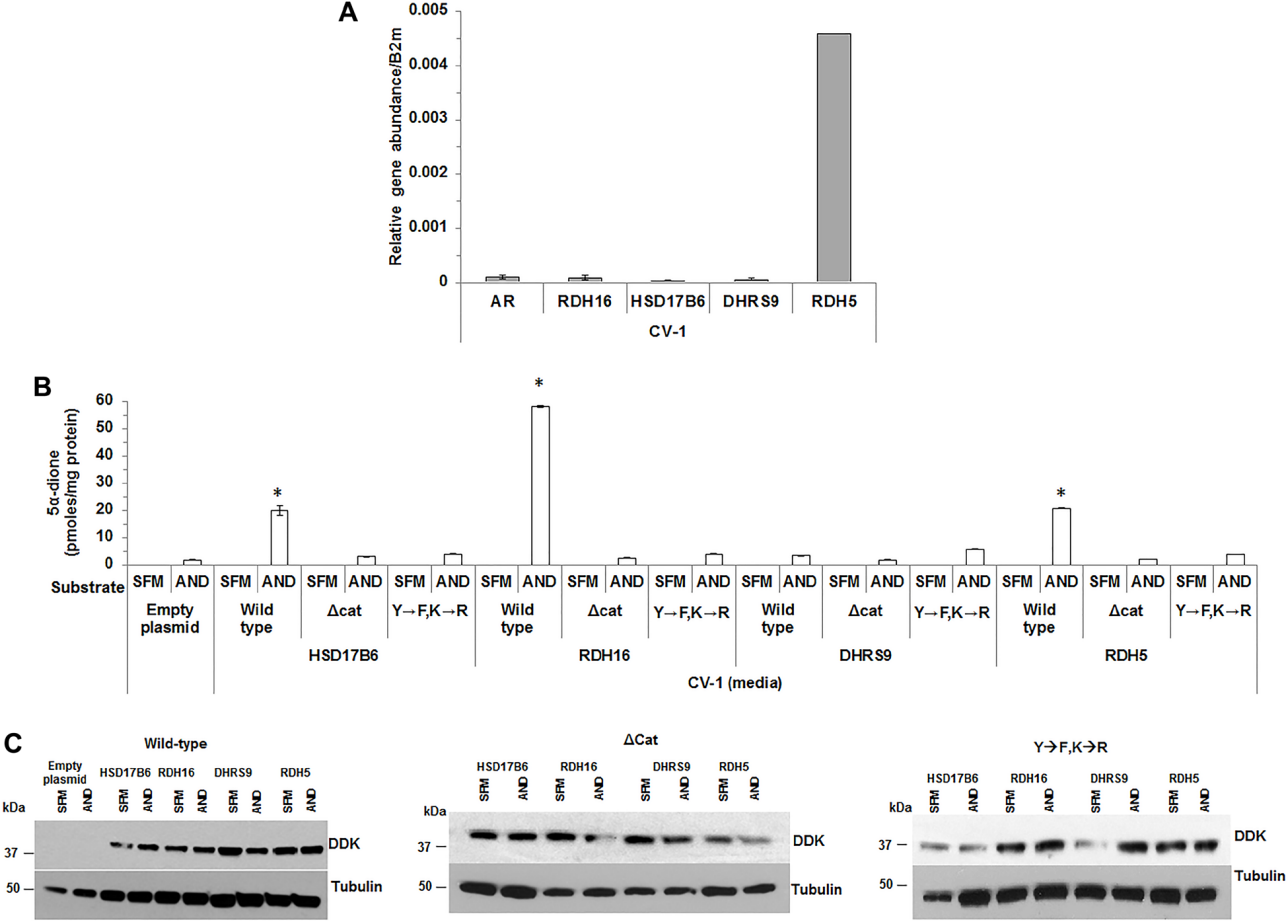
Mutation of conserved residues impaired 3α-oxidoreductase activity CV-1 cells were analyzed for 3α-oxidoreductase gene expression (**A**). 5α-dione levels were measured in CV-1 cells that transiently expressed wild-type HSD17B6, RDH16, DHRS9 or RDH5 wild-type 3α-oxidoreductases catalytic site deletion mutant (∆cat) or double mutant (Y→F, K→R) (**B**). Transient 3α-oxidoreductase expression was confirmed using western blotting with DDK antibody (**C**). Western blot images were cropped to conserve presentation space. Data were presented as the mean +/- SEM. Mean media androgen levels were compared between AND or SFM treated CV-1 cells using one-sided Tukey-Kramer adjusted Welch-Satterthwaite *T*-tests; corresponding *P-*values were listed in Supplementary Table 6. Media androgen levels of AND treated CV-1 cells were modeled as a function of enzyme (HSD17B6, RDH16, DHRS9 or RDH5, or empty plasmid), plasmid type (wild-type, Y→F, K→R or ∆cat 3α-oxidoreductases) and their interaction using a GLM. Mean levels were compared among plasmid types for each 3α-oxidoreductase, and among 3-oxidoreductases and the empty plasmid for each plasmid type using Tukey-Kramer adjusted post-hoc tests. *P-*values were reported in Supplementary Table 8. ^*^p < 0.05.

qRT-PCR confirmed that CV-1 cells had low endogenous AR and 3α-oxidoreductase mRNA levels that suggested RDH5 converted AND to 5α-dione in control CV-1 cells (Figure 4A). 3α-oxidoreductase protein expression was not detected using western blotting (data not shown). Therefore, despite low expression of RDH5, CV-1 cells were used to express wild-type or mutant 3α-oxidoreductases to evaluate the effect of the mutations on the activity of 3α-oxidoreductases.

CV-1 cells treated with AND produced only a small amount of 5α-dione that was measurable only in the media (Figure 4B). T or DHT was not detected in the media or CV-1 cell pellets. Therefore, subsequent experiments measured only androgens in media. CV-1 cells that expressed wild-type HSD17B6, RDH16 or RDH5 and were treated with AND produced 5α-dione at levels higher than levels observed in media from CV-1 cells with empty plasmid (Supplementary Table 8). ∆cat or the Y→F,K→R mutations reduced 5α-dione levels to background. Wild-type DHRS9 activity was impaired in CV-1 cells, which suggested CV-1 cells possessed inhibitory mechanisms that interfered with DHRS9 activity (Figure 4B). 5α-dione levels were not increased in CV-1 cells that expressed Y→F,K→R or ∆cat 3α-oxidoreductase mutants. 3α-oxidoreductase enzyme expression was verified using western blot (Figure 4C). The findings suggested that Y176 (Y175) and K180 (K179) were critical residues for enzyme activity for three of the four 3α-oxidoreductases.

### 3α-oxidoreductase expression in AS-BP and CaP specimens from research subjects treated with finasteride

**Figure 5:**
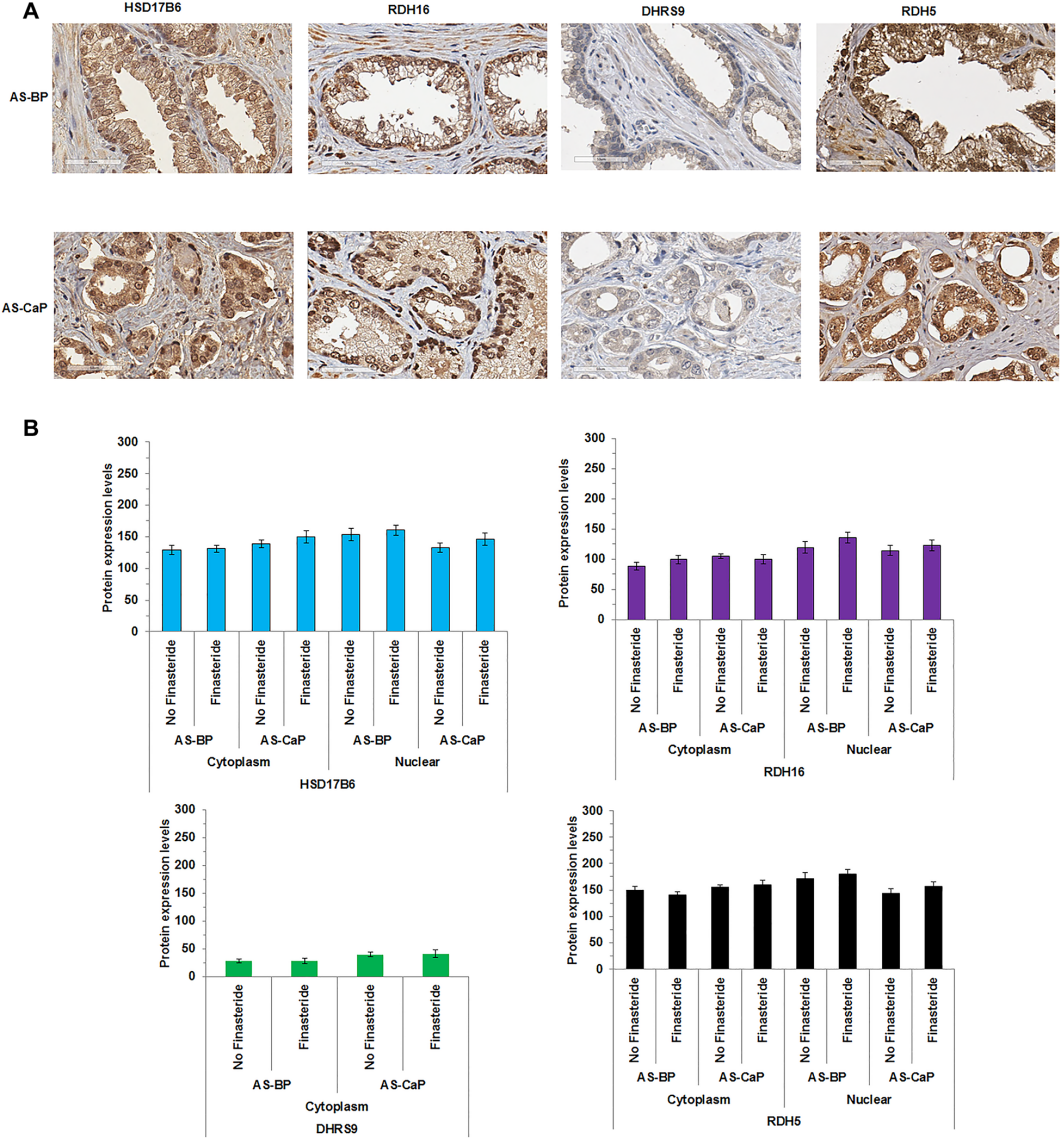
3α-oxidoreductases were expressed in AS-BP and AS-CaP tissues from research subjects treated with finasteride HSD17B6, RDH16 and RDH5 (**A**) were detected using IHC in androgen stimulated-benign prostate (AS-BP) and CaP (AS-CaP) tissues. Visual scoring showed 3α-oxidoreductase expression levels did not change with or without finasteride treatment (**B**). Data were presented as mean +/- SEM. Protein expression levels were modeled as a function of treatment (finasteride and no finasteride), tissue type (AS-BP or AS-CaP), their interaction, and a random subject effect using a LMM. Mean levels were compared between treatments within each tissue type, and between tissue types for each treatment using Tukey-Kramer adjusted tests about the least-square means. *P-*values were reported in Supplementary Table 9.

To address the clinical relevance of the 3α-oxidoreductases, LC-MS/MS was applied to tissues obtained from a randomized, double-blind, placebo-controlled clinical trial of selenium supplementation and finasteride treatment of research subjects with CaP for two to four weeks prior to radical prostatectomy (Selenium and Finasteride Pre-Treatment Trial, I104607). Research subjects who received finasteride alone or in combination with selenium were compared to research subjects who received selenium alone or placebo. Research subjects treated with finasteride had decreased CaP tissue levels of DHT in both benign and malignant macro-dissected samples, although DHT levels remained sufficient to activate AR (data not shown). TMAs generated from the clinical trial were sectioned and analyzed using IHC. HSD17B6, RDH16, DHRS9 and RDH5 were expressed in AS-BP and AS-CaP (Figure 5A–5B). HSD17B6, RDH16, DHRS9 and RDH5 expression levels and subcellular localization were similar to the 72 research subjects’ specimens analyzed previously (Figure 1D–1E). The data suggested that DHT synthesis persisted in spite of finasteride inhibition of SRD5A either from incomplete inhibition of SRD5A or from backdoor DHT synthesis.

### Combination dutasteride and 3α-oxidoreductase mutants decreased DHT greater than dutasteride alone

Primary backdoor DHT synthesis may facilitate CaP resistance to 5α-reductase inhibition. Therefore, simultaneous inhibition of the terminal steps of the frontdoor and primary backdoor pathways could lower DHT levels more effectively than targeting either terminal step alone. LAPC-4 cells exhibited SRD5A activity and dutasteride treatment decreased LAPC-4 DHT levels [19]. LAPC-4 cells also were capable of backdoor DHT synthesis using 3α-oxidoreductases to convert DIOL to DHT (Figure 3A). Therefore, LAPC-4 cells were used to test the effect of inhibition of the terminal steps of the frontdoor and primary backdoor androgen pathways. Dutasteride was used to inhibit SRD5A activity and block the frontdoor pathway. Activity impairing mutants were used to block enzymatic activity in the primary backdoor pathway, since no effective inhibitors are available for the four 3α-oxidoreductases.

**Figure 6:**
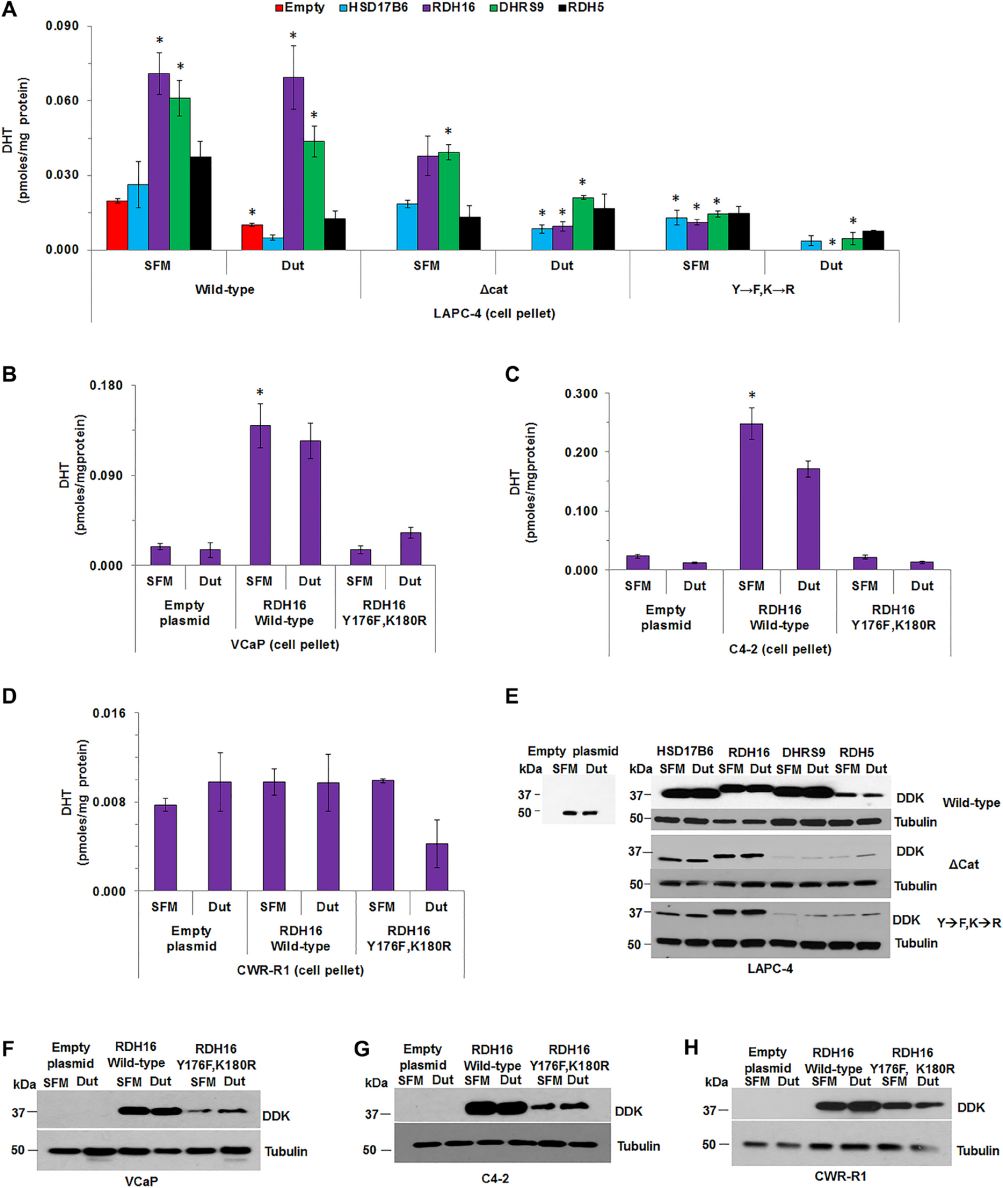
The combination of dutasteride and 3α-oxidoreductase mutants decreased DHT levels greater than dutasteride alone The effect of dutasteride on DHT levels was determined in LAPC-4 cells that transiently overexpressed wild-type, ∆cat or Y→F, K→R enzymes. (**A**) DHT levels were measured in VCaP, C4-2 and CWR-R1 cells transfected with empty plasmid or over-expressing plasmids of wild-type RDH16 or RDH16 Y176F,K180R (**B**–**D**). Western blot analysis using DDK antibody confirmed transiently expressed enzymes in LAPC-4, VCaP, C4-2 and CWR-R1 (**E**–**H**). Western blot images were cropped to conserve presentation space. Data were presented as mean +/- SEM. DHT was modeled as a function of treatment (dutasteride or SFM), enzyme (HSD17B6, RDH16, DHRS9 or RDH5) or empty plasmid, and plasmid type (wild-type, ∆cat or Y→F, K→R) using a GLM. Mean DHT levels were compared between dutasteride and SFM treated LAPC-4 cells using one-sided Tukey-Kramer adjusted tests about the least-square means. Mean levels were compared among LAPC-4 cells that expressed HSD17B6, RDH16, DHRS9 or RDH5, or empty plasmid, for each 3α-oxidoreductase-treatment combination using Tukey-Kramer adjusted tests about the least square means. *P*-values were reported in Supplementary Table 10. ^*^p < 0.05.

DHT levels were higher in LAPC-4 cell pellets that overexpressed RDH16 (*p* < 0.001) or DHRS9 (*p* < 0.001) compared to LAPC-4 cell pellets with empty plasmid (Figure 6A [SFM alone]; Supplementary Table 10); no androgens were measurable in media. DHT levels were lower in dutasteride treated LAPC-4 cells with empty plasmid compared to SFM treated LAPC-4 cells with empty plasmid (Figure 6A; *p* = 0.041; Supplementary Table 10). No effect of dutasteride was observed in LAPC-4 cells that expressed wild-type RDH16 or DHRS9, which suggested that RDH16 or DHRS9 was sufficient for primary backdoor DHT synthesis. LAPC-4 cells that expressed ∆cat of RDH5 or Y→F, K→R mutants of RDH16 or DHRS9 had lower DHT levels (RDH5, *p* = 0.046; RDH16, *p* = 0.006; DHRS9, *p* = 0.004) compared to LAPC-4 cells that expressed wild-type RDH16, DHRS9 or RDH5. DHT levels were lowered further by dutasteride treatment of LAPC-4 cells that expressed mutant RDH16 or DHRS9 compared to LAPC-4 cells treated with dutasteride alone. DHT levels were significantly lower in LAPC-4 cells that expressed RDH16-∆cat (*p* = 0.008), RDH16-Y176F,K180R (*p* = 0.004), DHRS9-∆cat (*p* = 0.029) or DHRS9-Y176F,K180R (*p* = 0.004) after dutasteride treatment compared to LAPC-4 cells that overexpressed wild-type RDH16 or DHRS9 (Figure 6A; Supplementary Table 10). Subsequent experiments focused on RDH16 because [1] wild-type RDH16 expression rendered dutasteride ineffective in LAPC-4 cells; and [2] DHT levels were lowered significantly by expression of RDH16-Y176F,K180R and dutasteride treatment.

Empty plasmid, or plasmids for over-expression of RDH16 wild-type or RDH16-Y176F, K180R were expressed into VCaP, LNCaP-C4-2 (C4-2) or CWR-R1 cells and DHT levels were measured using LC-MS/MS. DHT levels were similar in VCaP cells in SFM treated with or without dutasteride, which suggested that dutasteride treatment did not lower VCaP DHT levels (Figure 6B). The data were consistent with data reported previously [19]. Expression of wild-type RDH16 increased DHT levels (*p* = 0.031) that were affected minimally by dutasteride treatment. RDH16-Y176F,K180R expression resulted in DHT levels similar to VCaP cells that contained the empty plasmid. C4-2 cells produced measurable levels of DHT that were reduced by dutasteride treatment (*p* = 0.001; Figure 6C; Supplementary Table 10). Expression of wild-type RDH16 increased DHT levels (*p* = 0.001) that appeared to be reduced by dutasteride treatment. C4-2 cells that expressed RDH16-Y176,K180R produced DHT levels similar to C4-2 cells with empty plasmid. RDH16 did not appear to increase DHT levels in CWR-R1 cells and dutasteride did not lower DHT levels of CWR-R1 cells that contained empty plasmid or expressed wild-type RDH16 (Figure 6D). DHT levels appeared lower when CWR-R1 cells that expressed RDH16-Y176F,K180R were treated with dutasteride.

Western blot analysis demonstrated wild-type, ∆cat or Y→F,K→R mutant 3α-oxidoreductase expression in the four CaP cell lines (Figure 6E–6H). LC-MS/MS revealed that dutasteride appeared to lower T levels in all LAPC-4 cells, except in LAPC-4 cells that expressed RDH5-∆cat (data available upon request). LNCaP cells did not have measurable DHT, and T was not detected after dutasteride treatment (data available upon request).

## DISCUSSION

The findings provide proof of principal that the catalytic site shared by HSD176, RDH16, DHRS9 and RDH5 plays a role in intracrine androgen metabolism. RDH16 or DHRS9 enhanced DHT synthesis in LAPC-4 cells, which was consistent with previous reports from our group and others [5, 11–13, 34, 36, 37]. LAPC-4 and CV-1 LC-MS/MS data suggested 3α-oxidoreductase activity may be cell line-specific because CV-1 cells could not use DHRS9 to convert AND to 5α-dione and LAPC-4 cells could not use HSD17B6 to convert DIOL to DHT. However, HSD17B6 has been shown to convert DIOL to DHT in VCaP and LNCaP cell lines [38]. LC-MS/MS revealed that all four 3α-oxidoreductases converted AND to 5α-dione. Mutation of the common catalytic amino acids diminished 3α-oxidoreductase activity, which impaired CaP cells’ DHT synthesis using the primary backdoor pathway.

The current study is novel because the data showed that 3α-oxidoreductases are expressed in specimens of AS-BP, AS-CaP and CRPC, and AS-BP and AS-CaP after finasteride treatment. The primary backdoor pathway may facilitate DHT synthesis to overcome abiraterone treatment for CRPC and finasteride treatment for benign prostate enlargement or CaP chemoprevention. LC-MS/MS data provided evidence that all four 3α-oxidoreductases have similar enzymatic activity and that amino acid residues Y176/175 and K180/179 are essential for conversion of DIOL to DHT or AND to 5α-dione. The combination of dutasteride treatment and 3α-oxidoreductase mutation decreased DHT levels more effectively than dutasteride or 3α-oxidoreductase mutants alone.

The studies demonstrated that 3α-oxidoreductase expression may provide AS-BP, AS-CaP and CRPC with a mechanism for resistance to SRD5A inhibitors that primarily block frontdoor DHT synthesis.

A potential limitation of the study is that experiments could not be performed using endogenous 3α-oxidoreductases because enzyme expression was so low in CaP cell lines. Therefore, 3α-oxidoreductases were expressed transiently in CaP and CV-1 cells. Expression variability in cell-to-cell expression was a potential complication in cells that expressed 3α-oxidoreductases transiently. However, western blot analysis showed expression levels were consistent among wild-type enzymes. Western blots also showed 3α-oxidoreductase mutant expression was lower than the expression of wild-type 3α-oxidoreductases, which suggested amino acid substitutions may impair post-translational modification. However, sufficient enzyme was present based on the reproducibility of the replicates and differences in androgen metabolism observed among CaP cells transfected with control, wild-type or mutant 3α-oxidoreductases.

LC-MS/MS analyses were consistent across nine analytical runs conducted over a twenty month period. Sample generation, LC-MS/MS sample preparation and analysis improved over twenty months, which led to reduced replicate variability observed in later experiments. Reduced replicate variability explains why DHT levels produced from SFM only treated LAPC-4 cells that expressed DHRS9 were significantly higher than LAPC-4 cells with empty plasmid in Figure 6A, but not Figure 3A. Results were reliable over time and across replicates because the extracted calibration standards and plasma quality controls used to control and assess the quality of each LC-MS/MS analysis had an overall mean accuracy of 100% and 97.9%, respectively, for the androgens analyzed in CaP cell pellets and media over nine independent sets of experiments.

The LC-MS/MS method used for these studies was limited to six androgens and therefore, glucuronidated or sulfated androgens or androgen metabolites with activity in the glucocorticoid pathway were not measured. Cellular uptake and export of androgens and their metabolites were not considered [39]. The LC-MS/MS method could not measure DIOL because DIOL did not display a mass spectrum sufficiently different from other androgens. Therefore, AND conversion to 5α-dione was used to measure individual enzyme activity for the expression studies in CV-1 cells. The studies suggested that the conversion of AND to 5α-dione may provide an opportunity to use the secondary backdoor pathway to produce DHT [9].

The combination of dutasteride treatment and 3α-oxidoreductase mutation showed DHT levels were suppressed for 12 h. Clinical effectiveness will require longer studies of any potential inhibitor of the four 3α-oxidoreductases alone or in combination with dutasteride.

Taken together, the data [1] show that 3α-oxidoreductases are expressed in AS-BP, AS-CaP and CRPC; [2] demonstrate the catalytic residues necessary for the terminal step of the primary backdoor pathway, DIOL to DHT, are essential to all four 3α-oxidoreductases; and [3] provide evidence that combined SRD5A and 3α-oxidoreductase blockade lowered DHT levels more effectively than inhibition of either enzyme family alone. Inhibitors against the 3α-oxidoreductases are not yet available for clinical use and need to be identified. A new treatment strategy to block the key enzymatic steps of the frontdoor and primary and secondary backdoor pathways may decrease DHT levels more effectively than ADT alone. Further reduction of tissue DHT by inhibiting the last step of intracrine DHT synthesis should improve the clinical response to ADT or induce re-remission of CRPC and improve survival of men with advanced CaP.

## MATERIALS AND METHODS

### Experimental models

Human male CaP lines LAPC-4 [40], LNCaP [41], PC-3 cells (ATCC, Manassas, VA) and LNCaP-C4-2 (C4-2) cells [42, 43] were cultured in RPMI 1640 (Mediatech, Inc., Manassas, VA). CWR-R1 cells [44] were cultured using Richter’s Improved media (Corning, NY). CV-1 monkey kidney cells, DU145 [45] and VCaP cells (ATCC) were cultured in DMEM (Corning). RPMI and DMEM media were supplemented with 10% fetal bovine serum (FBS, Corning) and 2 mM glutamine (Corning). CWR-R1 cells were cultured in Richter’s Improved Media (Corning) supplemented with 1% epidermal growth factor (Thermo Fisher Scientific, Waltham, MA), 1% insulin-transferrin-sodium selenite supplement (Roche, Indianapolis, IN), 1% nicotinamide (Calbiochem, Billerica, MA) and 2% FBS. Androgen-dependent CWR22 [46] and ADT-recurrent CWR22 (rCWR22) [47] human CaP xenografts were propagated in immunocompromised nude mice.

All cell lines and xenografts were authenticated using genomic profiling in the RPCI Genomics Shared Resource. DNA profiles were acquired using 15 short tandem repeat (STR) loci and an amelogenin gender-specific marker. Test and control samples were amplified using the AmpFLSTR^®^ Identifiler^®^ Plus PCR Amplification Kit (Thermo Fisher Scientific, Waltham, MA) using the Verti 96-well Thermal Cycler (Applied Biosystems, Foster City, CA) in 9600 Emulation Mode (initial denature: 95°C 11 min, 28 cycles of denature: 94°C 20 sec and anneal/extend: 59°C 3 min, final extension: 60°C 10 min and hold: 12°C). PCR products were evaluated using the 3130xl Genetic Analyzer (Applied Biosystems) and analyzed using GeneMapper v4.0 (Applied Biosystems). Eight of the 15 STRs and amelogenin from the DNA profile for the cell lines were compared to the ATCC STR database and the DSMZ combined Online STR Matching Analysis databases. All matches above 80% were considered the same lineage.

### Method details

#### COBALT

The COBALT protein sequence alignment tool [48] was accessed using the National Center for Biotechnology (NCBI) website (http://www.ncbi.nlm.nih.gov/tools/cobalt/cobalt.cgi). Accession numbers for the 3α-oxidoreductases, HSD17B6, RDH16, DHRS9 and RDH5, were acquired using the NCBI protein database (http://www.ncbi.nlm.nih.gov/protein) and UniProtKB (http://www.uniprot.org/). Amino acid sequences of the four 3α-oxidoreductases were analyzed using COBALT and compared to other 3α-oxidoreductases and the SRD5A and CYP17 families to determine whether the catalytic site was conserved and specific to HSD17B6, RDH16, DHRS9 and RDH5.

### Site-directed mutagenesis

Site-directed mutagenesis was performed using the QuikChange^®^ Lightning Site-Directed Mutagenesis Kit (Strategene, Foster City, CA) and Strategene’s protocol. pCMV6-entry expression plasmids with C-terminal MYC-DDK tag encoded with HSD17B6, RDH16, DHRS9 or RDH5 were purchased from Origene (Origene, Rockville, MD). 3α-oxidoreductase primers (Integrated DNA Technologies, Coralville, IA) were used to generate ∆cat or Y→F, K→R 3α-oxidoreductase mutants (Supplementary Table 1). Plasmids were purified using PureYield Plasmid Miniprep System (Promega, Madison, WI) and sequenced at the Genomics Shared Resource. PCR for plasmid sequencing was performed using plasmid templates and Big Dye Terminator v3.1 Master Mix Kit (Life Technologies, Carlsbad, CA). PCR products were purified using Sephadex-G50 (Sigma-Aldrich, St. Louis, MO) into multiscreen HV plates (Thermo Fisher Scientific). Eluted samples were analyzed using 3130xl ABI Prism Genetic Analyzer. Sequence data were analyzed using Sequencing Analysis 5.2 software (Life Technologies).

### qRT-PCR

RNA extraction was performed using the RNeasy Plus Mini Kit (Qiagen, Valencia, CA). Samples generated from 9 cell lines were plated at 1 x 10^6^ cells/T25 cell culture flask (Corning). Cells were harvested using 0.05% trypsin and washed 3 times using Dulbecco’s phosphate buffered saline (PBS). PBS was removed and RLT lysis buffer was added to lyse the cell pellets. Frozen tissues from 2 xenografts (CWR22 and rCWR22) were Dounce homogenized in RLT lysis buffer. Lysates were passed through QIAshredder columns and RNA was extracted using RNeasy spin columns. Genomic DNA contamination was assessed using PCR and intron spanning GAPDH primers. Genomic DNA contamination was removed using a DNA-free DNA Removal Kit (Life Technologies). RNA was analyzed using PCR after DNase treatment to confirm genomic DNA was removed.

First strand complementary DNA (cDNA) was generated using 2 µg RNA and the High-Capacity cDNA Reverse Transcription Kit (10X RT Buffer, 10X Random Primers, 25X 100 mM deoxyNTP mix and 50 U/µL MultiScribe-Reverse Transcriptase; Applied Biosystems) and RNAse inhibitor in 10 µL reactions. The Primer Quest Primer Design Tool was used to design qRT-PCR primers (Integrated DNA Technologies) (Supplementary Table 1).

qRT-PCR reactions included 12.5 µL of SYBR Green PCR Master Mix (Applied Biosystems), 0.1 µL of 10 mM forward and reverse primers, 2.5 µL of 100 ng/µL cDNA (250 ng final concentration) and 9.8 µL distilled deionized (dd-H_2_O) for a final reaction volume 25 µL. Reactions were performed in 96-well plates. Gene expression was analyzed using 7300 Real Time System (Applied Biosystems). The qRT-PCR reaction parameters were 95°C for 30 sec, 60°C for 30 sec repeated 39 times, 95°C for 5 sec and melt curve 65°C-95°C. All procedures were performed with 3 technical replicates and 3 biological replicates. Cycle threshold (Ct) values were normalized against β2 microglobulin (B2M) that was selected based on unchanged B2M expression in SFM (data not shown). Ct values for negative RT controls and no template controls were reported as undefined. Relative gene abundance was calculated using 2^-(normalized Ct).

### Cell lysate and media preparation for LC-MS/MS analysis

LAPC-4, LNCaP, C4-2 or CWR-R1 cells were plated at 1.2 × 10^5^/well, VCaP cells at 2.5 × 10^5^ and CV-1 cells at 1 × 10^4^/well in 6-well tissue culture plates (Corning). Media and cell pellets from two 6-well plates were combined to generate 1 media and 1 cell pellet sample for LC-MS/MS analysis. LAPC-4, LNCaP, C4-2 or CWR-R1 cells were transfected using the Effectene Transfection Kit (Invitrogen, Grand Island, NY). Forty-eight h after transfection, growth media was removed and LAPC-4 cells were washed once with PBS (Corning). LAPC-4 cells were treated with serum-free complete media (SFM, Corning) alone or with 1 nM T, 20 nM DIOL 20 nM AND (Steraloids, Newport, RI) or 1 µM dutasteride (Selleckchem, Houston, TX) for 12 h. VCaP or CV-1 cells were transfected using X-tremeGene HP transfection reagent (Roche Diagnostics Corporation, Indianapolis, IN). After 48 h, growth media were aspirated, CV-1 cells were washed once with PBS and incubated in SFM alone or with 20 nM AND (Steraloids) for 12 h.

After 12 h treatment, media (total 24 mL) were collected in 50 mL conical tubes (Corning). Cells were released using trypsin and collected in 15 mL conical tubes (Corning). The cells were washed 3 times using PBS, re-suspended in 1 mL PBS and 5% of the cell suspension was removed for protein concentration measurement and western blot analysis. The remaining 95% of the cell suspension was centrifuged and the supernatant was removed and discarded. Cell pellets were stored at -80°C until analyzed using LC-MS/MS.

### LC-MS/MS

Cell pellet and media samples were analyzed over 9 runs for 5 androgens T, DHT, DHEA, ASD and AND using a validated LC-MS/MS method (Supplementary Methods and [49]). 5α-dione also was measured using this assay and data were included although results did not pass the strict validation acceptance criteria used for other androgens. Study samples were quantitated using aqueous-based spiked calibration standards and pre-spiked quality control samples prepared in 2X charcoal-stripped human postmenopausal female plasma (Bioreclamation, LLC, Westbury, NY). Performance data for calibrators, quality controls and calibration ranges were listed in Supplementary Table 2.

Androgen concentrations (pmoles/mg protein) in cell lysates measured using LC-MS/MS (ng/mL) were multiplied by the total volume of the lysate (1 mL), normalized by the total amount of protein of the cell pellet (mg), divided by the molecular weight of the androgen (ng/nmole), and converted to pmoles. Media androgen concentrations (ng/mL) were multiplied by the total volume of media (24 mL), normalized against total protein of the cell culture (mg), divided by the molecular weight of the androgen (ng/nmole) and converted to pmoles. Cell pellet and media androgen concentrations were reported combined in Results. Data from cell pellets or media and their statistical analysis provided upon similar results and are available on request. Experiments were performed in triplicate (*n* = 3).

### Western blotting

Cells removed from -80°C storage were resuspended in ubiquitin extraction lysis buffer (150 mM NaCl, 50 mM Tris-HCl, pH 7.4, 5 mM EDTA, 1% NP40, 0.5% sodium deoxycholate [all from Fisher, Pittsburg, PA]) and 0.1% SDS (Quality Biological, Gathersburg, MD). Halt Protease Inhibitor Cocktail (Sigma) was added to the ubiquitin lysis buffer just before cells were lysed. Cells were freeze-thawed three times and centrifuged at 14,000 x g for 15 min. Supernatants were transferred to clean microfuge tubes. Protein was quantified using the Protein Determination Kit (BioRad) and analyzed in flat bottom 96-well plates using an EL800 University Microplate Reader (BioTek Instruments) and KC Junior software (BioTek Instruments). SDS-polyacrylamide gel electrophoresis (PAGE) was performed using 4-15% Mini Trans-Blot cell (BioRad). Protein was transferred to Immuno-Blot PVDF membranes for Protein Blot (BioRad) and blocked in 5% milk in Tris-buffered saline with Tween 20 (TBST, Amersham Bioscience, GE Healthcare Bio-Sciences, Pittsburg, PA) for 30 min.

Membranes were incubated with DDK targeted antibody (Origene) 1:1000 overnight at room temperature. After incubation, blots were washed three times with TBST (Amersham Bioscience) for 10 min each. Washed blots were incubated with goat anti-mouse secondary antibody (Jackson ImmunoResearch Laboratories, West Grove, PA) 1:1000 for 1 h at room temperature. Blots were washed with TBST three times for 10 min each and protein expression was measured using the Pierce ECL Western Blotting Substrate (Life Technologies). Immunoblots were washed with TBST, blocked in 5% milk in TBST, reprobed for tubulin (1:1000 for 1 h at room temperature; Abcam, Cambridge, MA) and incubated with goat anti-rabbit secondary antibody (1:1000 for 1 h at room temperature; Jackson ImmunoResearch Laboratories).

### Tissue microarray (TMA) construction

Matched AS-BP and AS-CaP tissue specimens were collected from 36 patients who underwent radical prostatectomy. CRPC specimens were collected from 36 patients who underwent transurethral resection of the prostate for urinary retention from CaP that recurred during ADT. Specimens were collected between 1991 and 2011 at the Roswell Park Cancer Institute or the University of North Carolina at Chapel Hill, NC. TMAs were constructed (0.6 millimeter tissue cores) from formalin-fixed, paraffin-embedded donor blocks from each patient, which was guided by genitourinary pathologists. TMAs contained control tissue from lung, tonsil, liver, kidney, colon, spleen, cervix, thyroid, ovary, testis, myometrium and brain.

TMAs were constructed using the same process on tissues collected from a randomized, double-blind, placebo-controlled clinical trial of selenium supplementation and finasteride treatment of patients with CaP prior to robotic prostatectomy (Selenium and Finasteride Pre-Treatment Trial ID:NCT00736645). Forty-seven patients scheduled for radical prostatectomy were randomized into one of four treatment groups: placebo, finasteride, selenium or combination finasteride and selenium.

### IHC

TMA sections were de-paraffinized, rehydrated under an alcohol gradient and antigen retrieved using Reveal Decloaker (Biocare Medical, Concord, CA) for 30 min at 110°C and 5.5–6.0 psi. Sections were immunostained for the four 3α-oxidoreductases and AR as described (Supplementary Table 3). Enzymatic activity was assayed using 3,3’-Diaminobenzidine (Sigma-Aldrich) and sections were counterstained with hematoxylin (Vector Laboratories, Burlingame, CA). Sections were dehydrated and mounted using permanent mounting medium. Section images were collected using a Leica DFC0425C camera mounted on a Leica DMRA2 microscope (Leica Microsystems Inc., Buffalo Grove, IL). Protein expression was determined by three scorers who assessed the immunostain intensity and assigned values between 0 (no immunostain) and 3 (dark immunostain) for 100 cells per core that generated an average final score between 0 and 300 [50–52].

### Quantification and statistical analysis

IHC and qRT-PCR expression data were summarized graphically using mean and standard error. IHC experiments produced average scores between 0 and 300 for each tissue specimen from each patient in each TMA; qRT-PCR experiments had a sample size of *n* = 9 (three biological replicates analyzed using three technical replicates) per treatment group. Specific treatment groups details or changes in sample size for each experiment were listed in the figure legends.

IHC and qRT-PCR expression data, and androgen concentrations, were modeled as a function of tissue type (AS-BP, AS-CaP or CRPC), cell-line (VCaP, LNCaP, LAPC-4, C4-2, PC-3, DU145, CWR-R1, CWR22 or rCWR22), enzyme (HSD17B6, RDH16, DHRS9 or RDH5) or empty plasmid, treatment (SFM, T, DIOL, AND or dutasteride), expressed 3α-oxidoreductase (wild-type, Δcat or Y→F, K→R mutant enzymes), interaction terms and random replicate effects using general linear models (GLMs) or linear mixed models (LMMs). Factors, factor levels, interaction terms and random effects were included in each model depended on the specific experiment and research question that was addressed. Mean differences of interest were evaluated using Dunnett or Tukey-Kramer adjusted tests about the appropriate least square means. All tests were two-sided, unless stated otherwise. All model assumptions were verified graphically using quantile-quantile and residual plots, with Box-Cox transformations applied as appropriate. Additional details for experiment specific analyses were provided in the appropriate figure legends.

All analyses were conducted in SAS v9.4 (Cary, NC) at a nominal significance level of 0.05; therefore a *P*-value less than 0.05 denoted a statistical significant result. No randomization techniques were utilized and no observations were excluded from analysis.

## SUPPLEMENTARY MATERIALS FIGURES AND TABLES







## Abbreviations

(ASD): 4androstene-3α,17β-dione
(androstanediol; DIOL): 5androstane3α,17β-diol
(SRD5A): 5α-reductase
(Androsterone; AND): 5α-androstan-3α-ol-17-one
(5α-dione): 5α-androstan-3,17-dione
(HSD) 6 (HSD17B6): 17β-hydrosteroid dehydrogenase
(AR): Androgen receptor
(ADT): Androgen Deprivation Therapy
(AS-BP): Androgen-stimulated benign prostate
(AS-CaP): Androgen-stimulated prostate cancer
(B2M): β2 microglobulin
(CRPC): Castration-recurrent prostate cancer
(cDNA): Complementary DNA
(COBALT): Constraint-based Multiple Protein Alignment Tool
(Ct): Cycle threshold
(DHEA): Dehydroepiandrosterone
(DHRS9): Dehydrogenase/reductase family member 9
(RDH5): Dehydrogenase/reductase family member 5
(DHT): Dihydroteosterone
(GLMs): General linear models
(IHC): Immunohistochemistry
(LMMs): Linear mixed models
(LC-MS/MS): Liquid-chromatography tandem mass spectrometry
(LNCaP): LNCaP-RPCI
(C4-2): LNCaP-C4-2
(K): Lysine
(NCBI): National Center for Biotechnology
(PAGE): Polyacrylamide gel electrophoresis
(CaP): Prostate cancer
(qRT-PCR): Quantitative Real-Time Polymerase Chain Reaction
(RDH16): Retinol dehydrogenase 16
(SFM): Serum-free complete media
(STR): Short tandem repeat
(T): Testosterone
(TMA): Tissue microarray
(Y): Tyrosine
