# Supplementary material for: Inhibition of dihydrotestosterone synthesis in prostate cancer by combined frontdoor and backdoor pathway blockade
Source: Oncotarget. 2018 Jan 10;9(13):11227–42. doi: 10.18632/oncotarget.24107 (PMC5834294; doi:10.18632/oncotarget.24107)
Supplement: Supplementary file 1 [file oncotarget-09-11227-s001.pdf]

Inhibition of dihydrotestosterone synthesis in prostate cancer by combined frontdoor and backdoor pathway blockade

SUPPLEMENTARY MATERIALS

Liquid chromatography-tandem mass spectrometry

Cell pellets were resuspended in HPLC-grade water and vortexed or sonicated to disrupt cell membranes to obtain a homogeneous suspension. Samples were extracted using 0.25 ml quality control, plasma blank or sample in 0.75-2.0 mL HPLC-grade water, 0.1 ml internal standard (IS) solution (75.0/225 pg/mL d_3-T/d_3-DHT) and 4 mL methyl-tert-butyl ether (MTBE, Omnisolve®, EMD Milipore, Billerica, MA) in glass screw-top tubes. Calibrators were prepared using 50 µl spiking solution prepared in 75% methanol and added to the extraction tube. Either the entire 1 mL cell pellet suspension was added to the extraction tube and the original container rinsed with 25% methanol in water or samples were volume and compositionally corrected. Tubes were capped with Teflon-lined caps, vortexed, rotated 15 min and centrifuged using a Sorvall model RT6000B centrifuge (Thermo Scientific) at 2,800 rpm and 4°C for 15-30 min to separate liquid phases. The aqueous phase was frozen in a dry ice/acetone bath and MTBE layer was poured into a clean glass conical tube. MTBE was evaporated at 37°C with nitrogen and the residue was reconstituted with 60% methanol. The suspension was centrifuged using Heraeus Multifuge X3R centrifuge (Thermo Scientific) at 2,800 rpm and 4°C for 5 min to separate insoluble materials. An aliquot of the supernatant was injected.

LC-MS/MS analysis of the extracted samples was performed using a Prominence UFLC System (Shimadzu Scientific Instruments, Kyoto, Japan) a QTRAP® 5500 mass spectrometer (AB Sciex, Framingham, MA) with an electrospray ionization source and two 10-port switching valves (Model EPC10W Valco Instruments Co. Inc., Houston, TX,). The first switching valve was mounted in the column oven and was used to perform inline sample cleanup. The second valve functioned as a divert valve to switch the column eluent between waste and the mass spectrometer. Chromatographic separation was achieved using a Phenomenex® Luna® C18(2) column (part number 00F-4251-B0) preceded by a Phenomenex® SecurityGuard™ cartridge (C18, part number AJ0-4286). The HPLC column was maintained at 60°C and flow rate was 175 µL/min using a biphasic gradient. Mobile phase A was 65% methanol containing 400 µL 1 M ammonium formate and 65 µL concentrated formic acid per liter. Mobile phase B was 100% methanol containing 400 µL 1 M ammonium formate and 65 µL concentrated formic acid per liter.

Analytes were detected using multiple reaction monitoring in positive ion mode controlled by AB SCIEX Analyst® software, version 1.6.2 (AB Sciex). Mass spectrometer conditions were ion spray voltage 5,250 volts, turbo gas temperature 700 °C, gas 1 = 65, gas 2 = 60, curtain gas = 20, collision-associated dissociation gas medium and unit mass resolution for Q1 and Q3. Nitrogen was used for all gases and voltages for maximum parent/fragment ion pair intensities were optimized using direct infusion and flow injection analysis. Calibration curves were generated using analyte/IS area response ratios versus nominal concentrations (ng/mL) and weighted linear regressions with a weighting factor of 1/concentration.[2] The IS used for T, ASD and DHEA was d_3-T and d_3-DHT was used for DHT and AND. Back-calculated concentrations were generated using the formula $x = (y - b)/m$ where x is the back-calculated concentration, y is analyte/IS ratio, b is y-intercept and m is slope.

Calibrator and quality control acceptance criteria required all acceptable concentrations to have accuracy deviations ≤ 15% from the nominal concentration and relative standard deviation criteria (% RSD) ≤ 15%, except at the lower limit of quantitation (LLOQ), listed in Supplementary Table 2 which was allowed 20% deviation for both parameters. Values below the LLOQ (BLQ) were treated as 0.

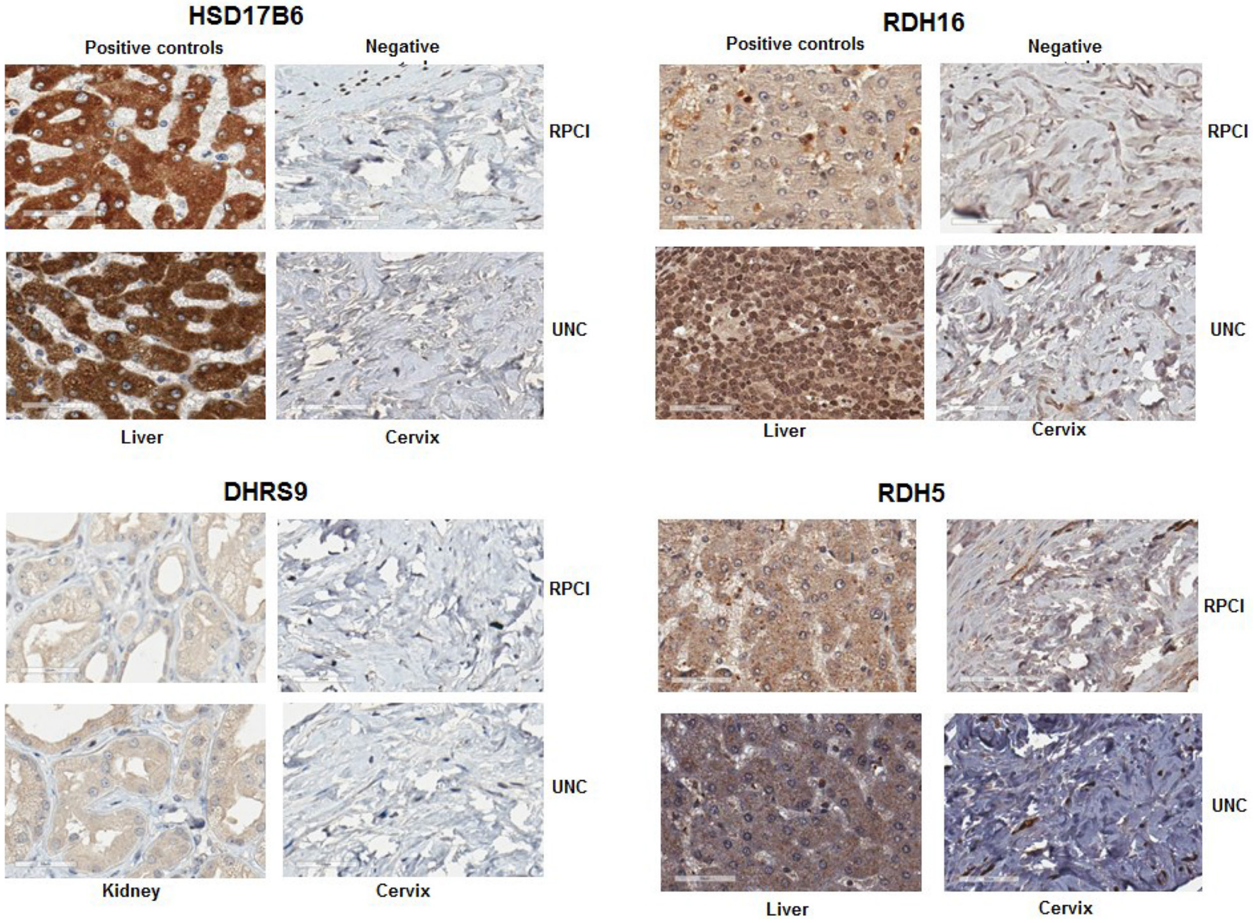

Supplementary Figure 1: IHC positive and negative controls for 3α-oxidoreductase antibodies used to immunostain TMA sections; Related to Figure 1. (RPCI = Roswell Park Cancer Institute TMA; UNC = University of North Carolina TMA.

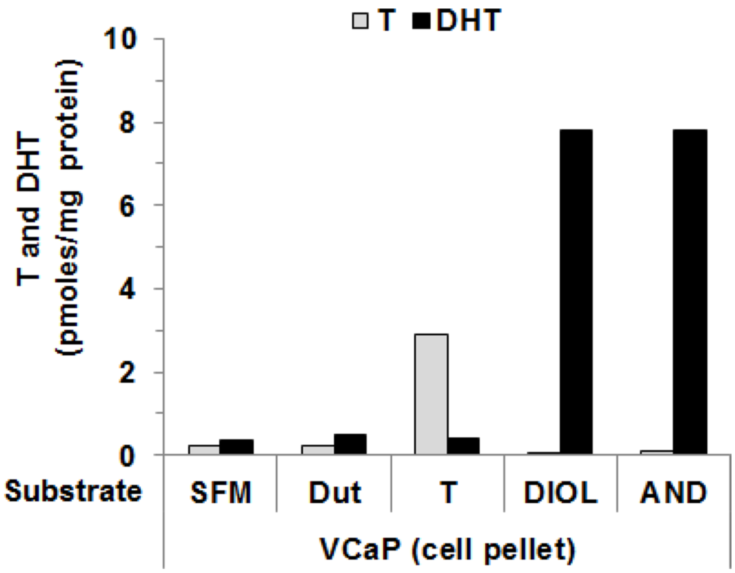

Supplementary Figure 2: Intracellular T and DHT levels from VCaP cells; Related to Figure 3. VCaP cells were treated for 12 h in SFM with or without 1 µM Dut, 1 nM T, 20 nM DIOL or 20 nM AND. VCaP cells were harvested, one cell pellet was analyzed for each condition and androgen levels were measured using LC-MS/MS as described in Methods. Dutasteride did not impair DHT synthesis without substrate addition. DHT levels increased when VCaP cells were treated with DIOL or AND and not T. The data suggested VCaP cells use backdoor metabolism to synthesize DHT.

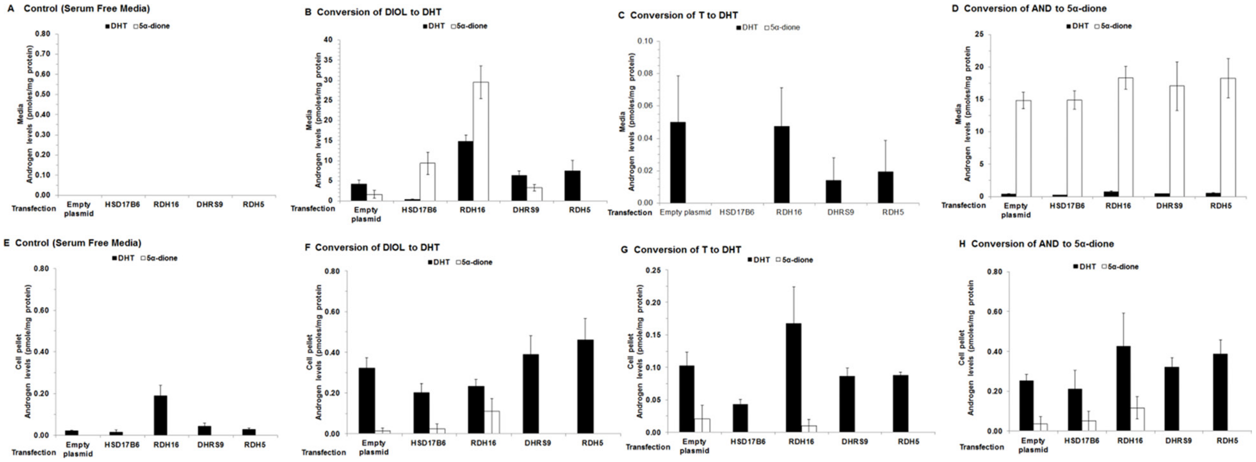

Supplementary Figure 3: Intracellular and media androgen levels for LAPC-4 cells transfected with empty plasmid or 3α-oxidoreductases; Related to Figure 3. Androgen levels were measured using LC-MS/MS from media (**A–D**) and cell pellets (**E–H**) of LAPC-4 cells transfected with empty plasmid or expression plasmids encoded with HSD17B6, RDH16, DHRS9 or RDH5. Cells were treated for 12 h in SFM or SFM with 1 nM T, 20 nM DIOL or 20 nM AND. Western blot analysis using DDK antibody was used to confirm enzyme expression (Figure 3E). Data were presented as mean +/– SEM. P-values generated from comparisons between 3α-oxidoreductases and LAPC-4 cells with empty plasmid were reported in S7.

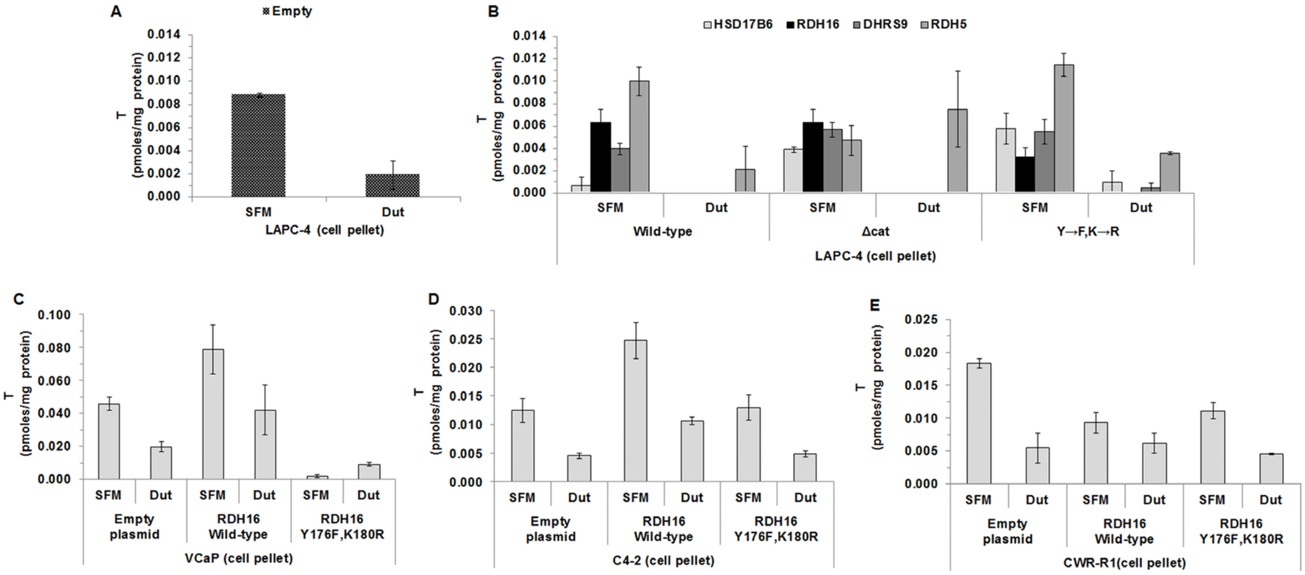

Supplementary Figure 4: Intracellular T levels after treatment with dutasteride or SFM; Related to Figure 6. LAPC-4, VCaP, C4-2 and CWR-R1 cells were transfected with empty, wild-type or Y176F,K180R RDH16, treated with SFM or SFM with dutasteride for 12 h and cell pellet androgen levels were measured using LC-MS/MS. T levels were reported for LAPC-4 (**A**), VCaP (**B**), C4-2 (**C**) and CWR-R1 (**D**).

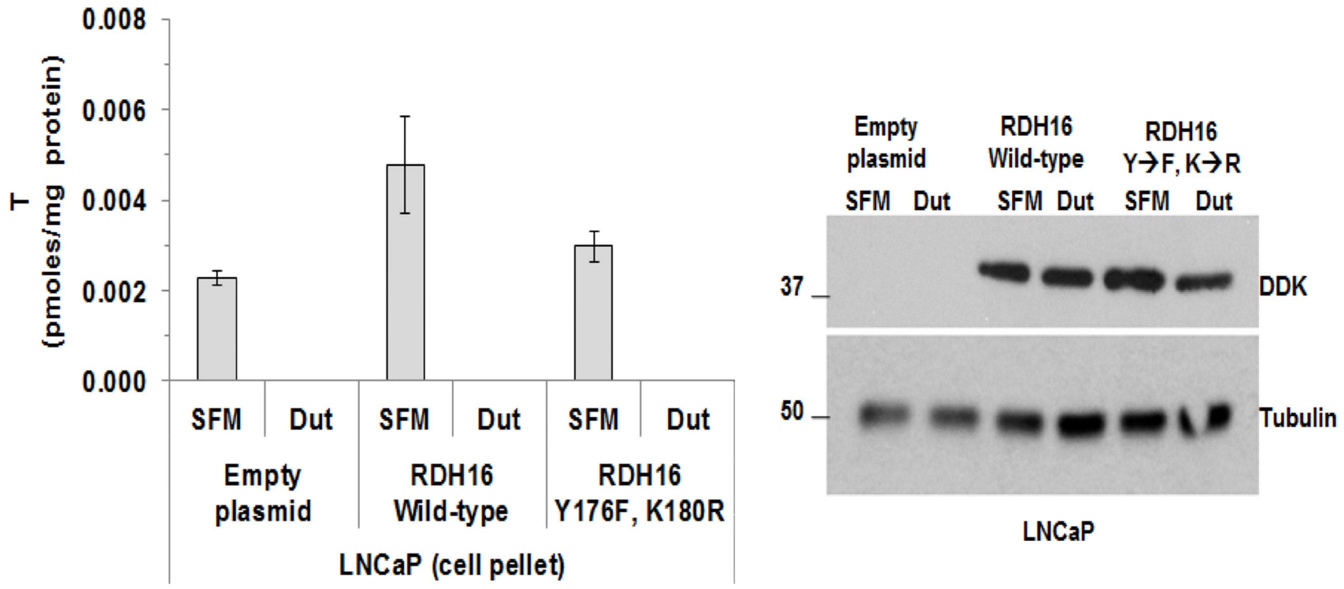

Supplementary Figure 5: Intracellular LNCaP T levels after treatment with dutasteride or SFM; Related to Figure 6. LNCaP cells were transfected with empty, wild-type or Y176F,K180R RDH16, treated with SFM or SFM with dutasteride for 12 h and cell pellet androgen levels were measured using LC-MS/MS. T levels for LNCaP (**A**) and western blot analysis for DDK tag (**B**).

Supplementary Table 1:

Site directed mutagenesis primers; Related to site-directed mutagenesis methods

HSD17B6	Δcat	FW	5′-gggaagagttgctttctttgtatatggagtggaagccttttc-3′
		RV	5′-gaaaaggcttccactccatatacaaagaaagcaactcttccc-3′
	Y176F,K180R	FW	5′-ggcttctgtgtctccaggtatggagtggaagcc-3′
		RV	5′-ggcttccactccatacctggagacacagaagcc-3′
RDH16	Δcat	FW	5′-gggtgtcactttttggttatggcgtggaagcctt-3′
		RV	5′-aaggcttccacgccataaccaaaaagtgacaccc-3′
	Y176F,K180R	FW	5′-gcttctgcatctccaggtatggcgtggaagc-3′
		RV	5′-gcttccacgccatacctggagatgcagaagc-3′
DHRS9	Δcat	FW	5′-cgccttgcaatcgttggatatgcagtggaaggtttc-3′
		RV	5′-gaaaccttccactgcatatccaacgattgcaaggcg-3′
	Y176F,K180R	FW	5′-aaggcttccacgccataaccaaaaagtgacaccc-3′
		RV	5′-aaccttccactgcatatctggatggagtaaagccc-3′
RDH5	Δcat	FW	5′-ctggcagccaatggttttggcctggaggcc-3′
		RV	5′-ggcctccaggccaaaaccattggctgccag-3′
	Y175F,K179R	FW	5′-gcttctgtgtctccagatttggcctggaggc-3′
		RV	5′-gcctccaggccaaatctggagacacagaagc-3′

Catalytic deletion (Δcat)

Primers used for qRT-PCR; Related to qRT-PCR methods

	Gene	Primer sequences	
1	HSD17B6	FW	5′-AGC ATG CTT CCT TTG GTG AGG AGA-3′
		RV	5′-TTC CCG TTC TGA AGT AGC CAG GTT-3′
2	RDH16	FW	5′-TGT GGT CAA CGT CTC CAG TGT CAT-3′
		RV	5′-AGA GAA GGC TTC CAC GCC ATA CTT-3′
3	DHRS9	FW	5′-GTC AAG AAA GCT CAA GGG AGA G-3′
		RV	5′-CCA CTG CAT ATT TGG ATG GAG TA-3′
4	RDH5	FW	5′-GGC GGG ATG TAG CTC ATT T-3′
		RV	5′-TTC TCC AGA CTC TCC AGG TT-3′
5	SRD5A1	FW	5′-TTC TGT ACC TGT AAC GGC TAT TT-3′
		RV	5′-GGG ATC TGT TAC CCA GTC ATC-3′
6	SRD5A2	FW	5′-CCT TCT GCA CTG GAA ATG GA-3′
		RV	5′-CAC CCA AGC TAA ACC GTA TGT-3′
7	SRD5A3	FW	5′-GGT CAT CTG CCC ATC AGT ATA AG-3′
		RV	5′-CCA AAT GGG ATC CTG TGG TTA-3′
8	AR (N-term)	FW	5′-CGG CTA ATG GGT GGA ATC TAA-3′
		RV	5′-GGT TAC ACC AAA GGG CTA GAA-3′
9	B2M	FW	5′-GAC TTG TAG AGA GAC AGG GTA GA-3′
		RV	5′-TAG GAG GGC TGG CAA CTT AG-3′

Supplementary Table 2: Related to LC-MS/MS methods

Compound	Calibrator Accuracy (%)		Calibrator Precision (%)	
	Mean	Range	Mean	Range
ASD	100	92.7-104	2.50	1.09-4.14
T	100	92.1-104	2.22	0.866-3.41
DHEA	100	98.5-101	4.20	2.75-7.79
DHT	100	92.5-104	2.89	1.18-5.11
AND	100	93.9-105	3.80	1.94-4.83
5α-dione	100	99.4-103	3.68	2.96-4.55

Compound	QC Accuracy (%)		QC Precision (%)	
	Mean	Range	Mean	Range
ASD	95.9	93.2-97.2	7.17	4.96-10.9
T	97.7	95.1-100	6.28	5.63-7.10
DHEA	96.8	95.6-98.2	7.67	5.79-9.06
DHT	101	96.9-103	7.63	6.26-8.72
AND	98.1	93.4-101	7.15	6.27-7.90
5α-dione	93.4	90.7-94.8	15.9	14.9-17.2

Compound	Serum calibration ranges	LLOQ
ASD	0.00625 - 3.75 ng/mL	0.00625 ng/mL
T	0.00625 - 3.75 ng/mL	0.00625 ng/mL
DHEA	0.200 - 7.50 ng/mL	0.200 ng/mL
DHT	0.0125 - 7.50 ng/mL	0.0125 ng/mL
AND	0.200 - 7.50 ng/mL	0.200 ng/mL
5α-dione	0.200 - 7.50 ng/mL	0.200 ng/mL

5α-dione did not pass standard acceptance criteria during assay validation (i.e., theoretical concentration ± 15% as recommended by FDA's Bioanalytical Guidance), but 5α-dione levels were reported since 5α-dione is an integral component of the androgen pathway under study. Overall performance statistics of the calibrators and quality controls for the nine analytical runs disclosed that six runs passed the normal ±15% criteria, one run passed at ±20%, one run passed at ±25% and one run passed at ±30%.

Supplementary Table 3: IHC antibodies and methods; Related to IHC methods

Target	HSD17B6	RDH16	DHRS9	RDH5	AR
Block step	None	Normal goat serum	Background Punisher	None	None
Primary antibody	HSD17B6	RDH16	DHRS9	RDH5	AR
Source	Abcam	Abcam	Abcam	Everest	DAKO
Primary ab catalog #	Ab88892	Ab89653	Ab89698	EB10078	M3562
Host	Mouse	Rabbit	Rabbit	Goat	Mouse
Primary ab dilution	1:100	1:200	1:600	1:100	1:100
Secondary ab	Goat anti-mouse	Goat anti-rabbit	Goat anti-rabbit	Rabbit anti-goat	Goat anti-mouse
Source	DAKO	DAKO	MACH 4 HRP polymer	DAKO	DAKO
Secondary ab catalog #	P0447	P0448	MRH534H	P0160	P0447
Secondary ab dilution	1:100	1:100	3-4 drops	1:100	1:100

Supplementary Table 4: Statistical comparisons of 3α-oxidoreductase cytosol and nuclear protein expression among tissue types; Related to Figure 1

		AS-BP vs AS-CaP	AS-BP vs CRCP	AS-CaP vs CRCP
HSD17B6	Cytosol	0.988	0.253	0.647
Nuclear	1.000	< 0.001	< 0.001	
RDH16	Cytosol	0.384	0.096	1.000
Nuclear	1.000	< 0.001	< 0.001	
DHRS9	Cytosol	0.502	0.501	0.095
RDH5	Cytosol	1.000	0.700	0.920
Nuclear	0.993	0.999	1.000	

Statistical comparisons of 3α-oxidoreductase protein expression between cytosol and nucleus; Related to Figure 1

		AS-BP	AS-CaP	CRPC
HSD17B6	Cytosol vs. Nuclear	0.994	0.449	0.807
RDH16	Cytosol vs. Nuclear	< 0.001	< 0.001	< 0.001
RDH5	Cytosol vs. Nuclear	0.023	0.890	1.000

Tukey-Kramer Adjusted *P*-values

Supplementary Table 5: Comparisons of enzyme expression among cell lines; Related to Figure 2.
see Supplementary Table 5.

Supplementary Table 6: Comparisons between treatment and SFM; Related to Figure 3 (Composite)

	Treatments compared	DIOL (B) vs. SFM (A)		T (C) vs. SFM (A)		AND (D) vs. SFM (A)	
	**Analyte**	**DHT**	**5α-dione**	**DHT**	**5α-dione**	**DHT**	**5α-dione**
Empty plasmid or enzyme	Empty plasmid	< 0.001	0.072	0.024	1.000	< 0.001	< 0.001
	HSD17B6	0.005	< 0.001	0.942	1.000	0.009	< 0.001
	RDH16	< 0.001	< 0.001	0.994	0.946	0.007	< 0.001
	DHRS9	< 0.001	< 0.001	0.173	1.000	< 0.001	< 0.001
	RDH5	< 0.001	1.000	0.069	1.000	< 0.001	< 0.001

Comparisons between 3α-oxidoreductases and empty plasmid for androgens; Related to Figure 3 (Composite)

	Treatment	SFM (A)		DIOL (B)		T (C)		AND (D)	
	Analyte	DHT	5α-dione	DHT	5α-dione	DHT	5α-dione	DHT	5α-dione
Comparisons	HSD17B6 vs. empty	0.556	-	< 0.001	0.022	0.124	0.469	0.270	1.000
	RDH16 vs. empty	0.008	-	0.022	0.003	0.670	0.880	0.292	0.664
	DHRS9 vs. empty	0.570	-	0.578	0.232	0.698	0.469	0.985	0.969
	RDH5 vs. empty	0.928	-	0.470	0.053	0.778	0.469	0.710	0.768

Dunnett Adjusted *P*-values

Supplementary Table 7: Comparisons between treatment and SFM (Media); Related to Fig. 3

**Treatments compared**		**DIOL (B) vs. SFM (A)**		**T (C) vs. SFM (A)**		**AND (D) vs. SFM (A)**	
Analyte		DHT	5α-dione	DHT	5α-dione	DHT	5α-dione
Empty plasmid or Enzyme	Empty	<0.001	0.081	0.278	1.000	<0.001	0.001
	HSD17B6	0.019	<0.001	1.000	1.000	0.049	<0.001
	RDH16	<0.001	<0.001	0.472	1.000	<0.001	<0.001
	DHRS9	<0.001	<0.001	0.833	1.000	<0.001	<0.001
	RDH5	<0.001	1.000	0.875	1.000	<0.001	<0.001

Comparisons between treatment and SFM (Cell pellet); Related to Fig. 3

Treatments compared		DIOL (B) vs. SFM (A)		T (C) vs. SFM (A)		AND (D) vs. SFM (A)	
Analyte		DHT	5α-dione	DHT	5α-dione	DHT	5α-dione
Empty plasmid or Enzyme	Empty	<0.001	0.072	0.002	1.000	<0.001	<0.001
	HSD17B6	0.005	<0.001	0.120	1.000	0.006	<0.001
	RDH16	0.869	<0.001	0.963	0.994	0.278	<0.001
	DHRS9	<0.001	<0.001	0.102	1.000	0.001	<0.001
	RDH5	<0.001	1.000	0.007	1.000	<0.001	<0.001

Comparisons between 3α-oxidoreductases and empty plasmid for androgens (Media); Related to Fig. 3

	Treatment	SFM (A)		DIOL (B)		T (C)		AND (D)	
	Analyte	DHT	5α-dione	DHT	5α-dione	DHT	5α-dione	DHT	5α-dione
Comparisons	HSD17B6 vs. empty	-	-	<0.001	0.024	-	-	0.072	1.000
	RDH16 vs. empty	-	-	0.366	0.004	-	-	0.086	0.671
	DHRS9 vs. empty	-	-	0.838	0.234	-	-	1.000	0.973
	RDH5 vs. empty	-	-	0.884	0.068	-	-	0.654	0.769

Comparisons between 3α-oxidoreductases and empty plasmid for androgens (Cell pellet); Related to Fig. 3

	Treatment	SFM (E)		DIOL (F)		T (G)		AND (H)	
	Analyte	DHT	5α-dione	DHT	5α-dione	DHT	5α-dione	DHT	5α-dione
Comparisons	HSD17B6 vs. empty	0.556	-	0.344	0.013	0.047	0.469	0.765	1.000
	RDH16 vs. empty	0.008	-	0.678	<0.001	0.409	0.880	0.718	0.664
	DHRS9 vs. empty	0.570	-	0.932	0.253	0.956	0.469	0.931	0.969
	RDH5 vs. empty	0.928	-	0.624	0.078	0.976	0.469	0.800	0.768

Tukey-Kramer Adjusted *P*-values.

Supplementary Table 8: Comparisons between AND and SFM treatments; Related to Figure 4

**Plasmid type**	**Enzyme**	**5α-dione**
Empty	-	0.376
Wild-type	HSD17B6	0.195
	RDH16	0.001
	DHRS9	0.039
	RDH5	< 0.001
Δcat	HSD17B6	0.039
	RDH16	0.042
	DHRS9	0.005
	RDH5	0.003
Y→F,K→R	HSD17B6	0.025
	RDH16	0.041
	DHRS9	0.005
	RDH5	0.001
Comparisons between wild-type, Δcat or Y→F,K→R; Related to Figure 4		
Enzyme	Comparison	5α-dione
HSD17B6	Wild-type vs. Δcat	0.015
	Wild-type vs. Y→F,K→R	0.026
RDH16	Wild-type vs. Δcat	0.001
	Wild-type vs. Y→F,K→R	< 0.001
DHRS9	Wild-type vs. Δcat	0.418
	Wild-type vs. Y→F,K→R	0.021
RDH5	Wild-type vs. Δcat	< 0.001
	Wild-type vs. Y→F,K→R	< 0.001
Comparisons between Enzyme and empty plasmid; Related to Figure 4		
Plasmid type	Comparison	5α-dione
Wild-type	HSD17B6 vs. empty	< 0.001
	RDH16 vs. empty	< 0.001
	DHRS9 vs. empty	0.158
	RDH5 vs. empty	< 0.001
Δcat	HSD17B6 vs. empty	0.048
	RDH16 vs. empty	0.002
	DHRS9 vs. empty	0.038
	RDH5 vs. empty	0.051
Y→F,K→R	HSD17B6 vs. empty	0.327
	RDH16 vs. empty	0.810
	DHRS9 vs. empty	1.000
	RDH5 vs. empty	0.993
Catalytic site deletion (Δcat), double mutation (Y→F,K→R)		
Tukey-Kramer Adjusted *P*-values		

Supplementary Table 9: Protein expression level comparison between non-finasteride and finasteride groups; Related to Figure 5

Tissue	Compartment	Treatments compared	HSD17B6	RDH16	RDH5	DHRS9	AR
AS-BP	Cytoplasm	No Finasteride - Finasteride	1	0.989	1.000	1.000	-
	Nuclear	No Finasteride - Finasteride	1	0.996	1.000	-	0.836
AS-CaP	Cytoplasm	No Finasteride - Finasteride	0.999	1.000	1.000	0.983	-
	Nuclear	No Finasteride - Finasteride	0.988	1.000	0.994	-	0.878
Protein expression level comparison by finasteride status and tissue type; Related to Figure 5							
Tissue	Compartment	Treatments compared	HSD17B6	RDH16	RDH5	DHRS9	AR
Cytoplasm	No Finasteride	AS-BP - CaP	1	0.882	1.000	0.241	-
	Finasteride	AS-BP - CaP	0.499	1.000	0.936	0.532	-
Nuclear	No Finasteride	AS-BP - CaP	0.155	1.000	0.669	-	0.999
	Finasteride	AS-BP - CaP	0.983	1.000	0.779	-	0.113
Tukey-Kramer Adjusted *P*-values							

Supplementary Table 10: Comparisons between dutasteride and SFM treated CaP cell lines; Related to Figure 6. See Supplementary_Table_10.
