# Supplementary material for: Inhibition of dihydrotestosterone synthesis in prostate cancer by combined frontdoor and backdoor pathway blockade
Source: Oncotarget. 2018 Jan 10;9(13):11227–42. doi: 10.18632/oncotarget.24107 (PMC5834294; doi:10.18632/oncotarget.24107)
Supplement: Supplementary file 2 [file oncotarget-09-11227-s002.docx]

| **Table S5: Comparisons of enzyme expression among cell lines. Related to Fig; 2** | | | | | | |
| --- | --- | --- | --- | --- | --- | --- |
| **VCaP**  **vs. LNCaP** | **VCaP**  **vs. LAPC-4** | **VCaP**  **vs. C4-2** | **VCaP**  **vs. PC-3** | |  |  |
| **<0.001** | **<0.001** | **<0.001** | **<0.001** | |  |  |
| **<0.001** | **<0.001** | **<0.001** | **<0.001** | |  |  |
| **0.998** | **0.278** | **1** | **<0.001** | |  |  |
| **<0.001** | **<0.001** | **<0.001** | **<0.001** | |  |  |
| **0.999** | **0.998** | **<0.001** | **0.109** | |  |  |
| **0.999** | **0.901** | **0.999** | **1** | |  |  |
| **<0.001** | **<0.001** | **0.575** | **1** | |  |  |
| **<0.001** | **<0.001** | **<0.001** | **<0.001** | |  |  |
|  |  |  |  | |  |  |
| **LNCaP**  **vs. LAPC-4** | **LNCaP**  **vs. C4-2** | **LNCaP vs.PC-3** | **LNCaP**  **vs. DU145** | |  |  |
| **<0.001** | **0.002** | **<0.001** | **<0.001** | |  |  |
| **<0.001** | **<0.001** | **<0.001** | **0.048** | |  |  |
| **0.69** | **1** | **<0.001** | **1** | |  |  |
| **0.001** | **0.667** | **<0.001** | **<0.001** | |  |  |
| **0.909** | **<0.001** | **0.384** | **<0.001** | |  |  |
| **0.999** | **1** | **0.999** | **0.987** | |  |  |
| **<0.001** | **<0.001** | **<0.001** | **<0.001** | |  |  |
| **0.084** | **0.988** | **0.003** | **0.003** | |  |  |
|  |  |  |  | |  |  |
| **LAPC-4**  **vs. C4-2** | **LAPC-4**  **vs. PC-3** | **LAPC-4**  **vs. DU145** | **LAPC-4**  **vs. CWR-R1** | |  |  |
| **0.998** | **0.028** | **1** | **<0.001** | |  |  |
| **0.746** | **0.333** | **<0.001** | **<0.001** | |  |  |
| **0.824** | **<0.001** | **0.39** | **0.595** | |  |  |
| **0.207** | **<0.001** | **<0.001** | **<0.001** | |  |  |
| **<0.001** | **0.016** | **<0.001** | **<0.001** | |  |  |
| **0.998** | **0.864** | **0.687** | **0.893** | |  |  |
| **<0.001** | **<0.001** | **<0.001** | **<0.001** | |  |  |
| **0.525** | **0.962** | **0.962** | **<0.001** | |  |  |
|  |  |  |  | |  |  |
| **C4-2**  **vs. PC-3** | **C4-2**  **vs. DU145** | **C4-2**  **vs. CWR-R1** | **C4-2**  **vs. CWR22** | |  |  |
| **0.003** | **0.993** | **<0.001** | **<0.001** | |  |  |
| **0.004** | **0.097** | **0.018** | **0.089** | |  |  |
| **<0.001** | **1** | **1** | **1** | |  |  |
| **<0.001** | **<0.001** | **<0.001** | **<0.001** | |  |  |
| **<0.001** | **<0.001** | **<0.001** | **<0.001** | |  |  |
| **0.998** | **0.98** | **0.999** | **0.031** | |  |  |
| **0.317** | **<.001** | **<0.001** | **<0.001** | |  |  |
| **0.051** | **0.051** | **<0.001** | **<0.001** | |  |  |
|  |  |  |  | |  |  |
| **PC-3**  **vs. DU145** | **PC-3**  **vs. CWR-R1** | **PC-3**  **vs. CWR22** | **PC-3**  **vs. rCWR22** | |  |  |
| **0.003** | **0.993** | **<0.001** | **<0.001** | |  |  |
| **<0.001** | **<0.001** | **<0.001** | **0.002** | |  |  |
| **<0.001** | **<0.001** | **<0.001** | **<0.001** | |  |  |
| **0.021** | **<0.001** | **<0.001** | **<0.001** | |  |  |
| **<0.001** | **<0.001** | **0.015** | **<0.001** | |  |  |
| **1** | **1** | **0.003** | **0.347** | |  |  |
| **<0.001** | **<.001** | **<0.001** | **<0.001** | |  |  |
| **1** | **<.001** | **<0.001** | **0.708** | |  |  |
|  |  |  |  | |  |  |
| **DU145**  **vs. CWR-R1** | **DU145**  **vs. CWR22** | **DU145**  **vs. rCWR22** |  | |  |  |
| **<0.001** | **<0.001** | **0.098** |  | |  |  |
| **0.999** | **1** | **0.146** |  | |  |  |
| **1** | **1** | **1** |  | |  |  |
| **<.001** | **<0.001** | **<0.001** |  | |  |  |
| **0.999** | **<0.001** | **<0.001** |  | |  |  |
| **1** | **0.001** | **0.192** |  | |  |  |
| **<0.001** | **<0.001** | **<.001** |  | |  |  |
| **<0.001** | **<0.001** | **0.708** |  | |  |  |
| **Tukey-Kramer adjustment *P-*values** | | | |  | |  |
