# Supplementary material for: Inhibition of dihydrotestosterone synthesis in prostate cancer by combined frontdoor and backdoor pathway blockade
Source: Oncotarget. 2018 Jan 10;9(13):11227–42. doi: 10.18632/oncotarget.24107 (PMC5834294; doi:10.18632/oncotarget.24107)
Supplement: Supplementary file 3 [file oncotarget-09-11227-s003.docx]

| **Table S10: Comparisons between dutasteride and SFM treated CaP cell lines; Related to Fig. 6** | | | | | | | | |
| --- | --- | --- | --- | --- | --- | --- | --- | --- |
| **Cell line** | | | **Plasmid type** | | | **Enzyme** | **DHT** | |
| **LAPC-4 (A)** | | | **Empty** | | | **-** | **0.041** | |
|  |  |  | **Wild-Type** | | | **HSD17B6** | **0.242** | |
|  |  |  |  |  |  | **RDH16** | **1.000** | |
|  |  |  |  |  |  | **DHRS9** | **0.315** | |
|  |  |  |  |  |  | **RDH5** | **0.094** | |
|  |  |  | **∆cat** | | | **HSD17B6** | **0.025** | |
|  |  |  |  |  |  | **RDH16** | **0.138** | |
|  |  |  |  |  |  | **DHRS9** | **0.045** | |
|  |  |  |  |  |  | **RDH5** | **1** | |
|  |  |  | **Y🡪F,K🡪R** | | | **HSD17B6** | **0.051** | |
|  |  |  |  |  |  | **RDH16** | **0.015** | |
|  |  |  |  |  |  | **DHRS9** | **0.159** | |
|  |  |  |  |  |  | **RDH5** | **0.157** | |
| **VCaP (B)** | | | **Empty** | | | **-** | **0.165** | |
|  |  |  | **Wild-Type** | | | **RDH16** | **0.029** | |
|  |  |  | **Y176F,K180R** | | | **RDH16** | **1.000** | |
| **C4-2 (C)** | | | **Empty** | | | **-** | **0.017** | |
|  |  |  | **Wild-Type** | | | **RDH16** | **0.151** | |
|  |  |  | **Y176F,K180R** | | | **RDH16** | **0.108** | |
| **CWR-R1 (D)** | | | **Empty** | | | **-** | **0.814** | |
|  |  |  | **Wild-Type** | | | **RDH16** | **0.988** | |
|  |  |  | **Y176F,K180R** | | | **RDH16** | **0.126** | |
| **Comparisons among wild-type, ∆cat and Y🡪F,K🡪R mutants; Related to Fig. 6B** | | | | | | | | |
|  | **Enzyme** | | | **Treatment** | **Enzyme Comparison** | | | **DHT** |
| **LAPC-4 (A)** | **HSD17B6** | | | **SFM** | **Wild-type vs. ∆cat** | | | **0.648** |
|  |  |  |  |  | **Wild-type vs. Y🡪F,K🡪R** | | | **0.326** |
|  | **RDH16** | | | **SFM** | **Wild-type vs. ∆cat** | | | **0.052** |
|  |  |  |  |  | **Wild-type vs. Y🡪F,K🡪R** | | | **0.006** |
|  | **DHRS9** | | | **SFM** | **Wild-type vs. ∆cat** | | | **0.057** |
|  |  |  |  |  | **Wild-type vs. Y🡪F,K🡪R** | | | **0.004** |
|  | **RDH5** | | | **SFM** | **Wild-type vs. ∆cat** | | | **0.046** |
|  |  |  |  |  | **Wild-type vs. Y🡪F,K🡪R** | | | **0.055** |
|  | **HSD17B6** | | | **Dut** | **Wild-type vs. ∆cat** | | | **0.362** |
|  |  |  |  |  | **Wild-type vs. Y🡪F,K🡪R** | | | **0.846** |
|  | **RDH16** | | | **Dut** | **Wild-type vs. ∆cat** | | | **0.008** |
|  |  |  |  |  | **Wild-type vs. Y🡪F,K🡪R** | | | **0.004** |
|  | **DHRS9** | | | **Dut** | **Wild-type vs. ∆cat** | | | **0.029** |
|  |  |  |  |  | **Wild-type vs. Y🡪F,K🡪R** | | | **0.004** |
|  | **RDH5** | | | **Dut** | **Wild-type vs. ∆cat** | | | **0.735** |
|  |  |  |  |  | **Wild-type vs. Y🡪F,K🡪R** | | | **0.649** |
| **VCaP (B)** | **RDH16** | | | **SFM** | **Wild-type vs. Y🡪F,K🡪R** | | | **0.005** |
|  | **RDH16** | | | **Dut** | **Wild-type vs. Y🡪F,K🡪R** | | | **0.910** |
| **C4-2 (C)** | **RDH16** | | | **SFM** | **Wild-type vs. Y🡪F,K🡪R** | | | **0.001** |
|  | **RDH16** | | | **Dut** | **Wild-type vs. Y🡪F,K🡪R** | | | **<0.001** |
| **CWR-R1 (D)** | **RDH16** | | | **SFM** | **Wild-type vs. Y🡪F,K🡪R** | | | **0.981** |
|  | **RDH16** | | | **Dut** | **Wild-type vs. Y🡪F,K🡪R** | | | **0.355** |
| **Comparisons between 3α-oxidoreductases and empty plasmid** | | | | | | | | |
|  | | **Enzyme** | | **Treatment** | **Comparison** | | | **DHT** |
| **LAPC-4 (A)** | | **HSD17B6** | | **SFM** | **Empty vs. Wild-type** | | | **1.000** |
|  |  |  |  | **Dut** | **Empty vs. Wild-type** | | | **1.000** |
|  |  |  |  | **SFM** | **Empty vs. ∆cat** | | | **1.000** |
|  |  |  |  | **Dut** | **Empty vs. ∆cat** | | | **1.000** |
|  |  |  |  | **SFM** | **Empty vs. Y🡪F,K🡪R** | | | **1.000** |
|  |  |  |  | **Dut** | **Empty vs. Y🡪F,K🡪R** | | | **1.000** |
|  |  | **RDH16** | | **SFM** | **Empty vs. Wild-type** | | | **<0.001** |
|  |  |  |  | **Dut** | **Empty vs. Wild-type** | | | **<0.001** |
|  |  |  |  | **SFM** | **Empty vs. ∆cat** | | | **0.550** |
|  |  |  |  | **Dut** | **Empty vs. ∆cat** | | | **1.000** |
|  |  |  |  | **SFM** | **Empty vs. Y🡪F,K🡪R** | | | **0.999** |
|  |  |  |  | **Dut** | **Empty vs. Y🡪F,K🡪R** | | | **0.992** |
|  |  | **DHRS9** | | **SFM** | **Empty vs. Wild-type** | | | **<0.001** |
|  |  |  |  | **Dut** | **Empty vs. Wild-type** | | | **0.002** |
|  |  |  |  | **SFM** | **Empty vs. ∆cat** | | | **0.398** |
|  |  |  |  | **Dut** | **Empty vs. ∆cat** | | | **0.985** |
|  |  |  |  | **SFM** | **Empty vs. Y🡪F,K🡪R** | | | **1.000** |
|  |  |  |  | **Dut** | **Empty vs. Y🡪F,K🡪R** | | | **1.000** |
|  |  | **RDH5** | | **SFM** | **Empty vs. Wild-type** | | | **0.552** |
|  |  |  |  | **Dut** | **Empty vs. Wild-type** | | | **1.000** |
|  |  |  |  | **SFM** | **Empty vs. ∆cat** | | | **1.000** |
|  |  |  |  | **Dut** | **Empty vs. ∆cat** | | | **1.000** |
|  |  |  |  | **SFM** | **Empty vs. Y🡪F,K🡪R** | | | **1.000** |
|  |  |  |  | **Dut** | **Empty vs. Y🡪F,K🡪R** | | | **1.000** |
| **VCaP (B)** | | **RDH16** | | **SFM** | **Empty vs. Wild-type** | | | **0.031** |
|  |  |  |  | **Dut** | **Empty vs. Wild-type** | | | **0.116** |
|  |  |  |  | **SFM** | **Empty vs. Y🡪F,K🡪R** | | | **0.229** |
|  |  |  |  | **Dut** | **Empty vs. Y🡪F,K🡪R** | | | **0.149** |
| **C4-2 (C)** | | **RDH16** | | **SFM** | **Empty vs. Wild-type** | | | **0.001** |
|  |  |  |  | **Dut** | **Empty vs. Wild-type** | | | **<0.001** |
|  |  |  |  | **SFM** | **Empty vs. Y🡪F,K🡪R** | | | **0.996** |
|  |  |  |  | **Dut** | **Empty vs. Y🡪F,K🡪R** | | | **0.993** |
| **CWR-R1 (D)** | | **RDH16** | | **SFM** | **Empty vs. Wild-type** | | | **0.251** |
|  |  |  |  | **Dut** | **Empty vs. Wild-type** | | | **0.878** |
|  |  |  |  | **SFM** | **Empty vs. Y🡪F,K🡪R** | | | **0.206** |
|  |  |  |  | **Dut** | **Empty vs. Y🡪F,K🡪R** | | | **0.212** |
| **Catalytic site deletion (∆cat), double mutation (Y🡪F,K🡪R)** | | | | | | | | |
| **Tukey-Kramer Adjusted *P-*values** | | | | | | | | |
